# Revision of the Neotropical water scavenger beetle genus *Tobochares* Short & García, 2007 (Coleoptera, Hydrophilidae, Acidocerinae)

**DOI:** 10.3897/zookeys.669.11773

**Published:** 2017-04-21

**Authors:** Alex T. Kohlenberg, Andrew Edward Z. Short

**Affiliations:** 1 Department of Ecology & Evolutionary Biology & Division of Entomology, Biodiversity Institute, University of Kansas, Lawrence, KS 66045, USA

**Keywords:** South America, Guiana Shield, taxonomy, new species

## Abstract

The genus *Tobochares* Short & García, 2007 is revised. A combination of morphological and molecular data provide support for ten distinct species. Five new species are described: *T. canaliculatus* **sp. n.** (Venezuela), *T. canthus* **sp. n.** (Venezuela), *T. emarginatus* **sp. n.** (Suriname), *T. kusad* **sp. n.** (Guyana), and *T. pallidus* **sp. n.** (Venezuela). All four preexisting species are redescribed. A tenth species, known from a single female from Venezuela, is left undescribed pending the collection of additional specimens. New collecting records are provided for *T. sulcatus* Short & García, 2007 and *T. kasikasima* Short, 2013. *Tobochares sipaliwini* Short & Kadosoe, 2011 is newly recorded from Guyana. All species are associated with seepage or wet rock habitats, although some species have also been found along the margins of streams that flow over rocky substrates. High-resolution images including scanning electron micrographs are provided, as well as a key to species and habitat photographs.

## Introduction

The genus *Tobochares* Short & García, 2007 was erected for a single unusual species found living under leaves on wet rock along a creek in southern Venezuela (Short and García 2007). In the decade that has elapsed since its description, additional fieldwork across the northern Guiana Shield has produced more than 400 additional specimens of the genus from Venezuela, Suriname, and Guyana. A few of these were described as they were identified (e.g. Short and Kadosoe 2011, Short 2013), and the genus has grown to contain a total of four species. A comprehensive morphological review of this material as well as DNA sequence data from the mitochondrial gene Cytochrome Oxidase I (COI) has uncovered six additional undescribed species, of which five are described herein. The sixth species is known only from a single partly disarticulated female, which we refrain from describing until additional material can be collected. The discovery of these new species has required a slight broadening of the concept of *Tobochares*, most notably that not all species have grooved elytra, and consequently the genus is here redescribed. We also provide detailed habitat information for all species, which appears to be narrowly restricted to rock seepages and associated habitats.

## Materials and methods

**Depositories of examined material.**

**CBDG** Center for Biological Diversity, University of Guyana, Georgetown

**MALUZ** Museo de Artrópodos de la Universidad del Zulia, Maracaibo, Venezuela (J. Camacho, M. García)

**MIZA** Museo del Instituto de Zoología Agrícola, Maracay, Venezuela (L. Joly)

**NZCS** National Zoological Collection of Suriname, Paramaribo (P. Ouboter, V. Kadosoe)

**SEMC** Snow Entomological Collection, University of Kansas, Lawrence, KS (A. Short)

**USNM** U.S. National Museum of Natural History, Smithsonian Institution, Washington, DC (C. Micheli).

**Morphological methods.** Specimens were examined using an Olympus SZX16 microscope (to 110× magnification). Specimens for dissection were relaxed in warm water, and their genitalia were removed and placed in glycerin on a glass slide, which was then viewed and imaged using an Olympus BX51 to 200× magnification. Genitalia were mounted beneath the specimens in microvials with glycerin. Scanning electron micrographs were taken by mounting specimens on carbon tape and coated in gold. Habitus photographs were taken with a Visionary Digital imaging system. All final images were created by stacking multiple individual photographs from different focal planes using the software Zerene Stacker. Morphological terminology largely follows Hansen (1991) except for the use of meso- and metaventrite instead of meso- and metasternum.

**Molecular methods.** Total genomic DNA was extracted from entire beetles using a DNeasy kit (Qiagen, Alameda, CA). All vouchers (Table 1) are deposited at the University of Kansas (Lawrence, USA). We selected specimens of each putative morphospecies from each locality for which we had specimens preserved in 100% ethanol; we did not have suitable material for two morphospecies (*T. canthus* and *T. canaliculatus*) which are thus not included in our molecular analyses. We used the COI primers and PCR protocols as given in Short and Fikáček (2013). Resulting DNA sequences were assembled and edited in Geneious R 8.0.5 (Biomatters, http://www.geneious.com/), which was also used to examine the raw pairwise distances between sequences. All new sequences are deposited in GenBank (see Table 1 for accession numbers). IQ-TREE 1.4.4 (Nguyen et al. 2015) was used to conduct a maximum likelihood analysis to infer phylogenetic relationships. The optimal model of substitution was selected using the Auto function in IQ-TREE 1.4.4; default settings were used for the analysis. In order to assess nodal support, we performed 1000 ultrafast bootstrap replicates (Minh et al. 2013). We included representatives of two related acidocerine genera (Short and Fikáček 2013) *Chasmogenus ruidus* Short, 2005 and *Globulosis flavus* Short, Garcia, & Giron, 2017 as outgroups to root the tree (GenBank accessions KC935240 and KY351811 respectively).

**Table 1.** List of specimens and GenBank accession numbers used in this study. All vouchers are deposited in SEMC.

Taxon	Extraction	Locality	Coordinates	GenBank Accession
*T. emarginatus*	SLE424	Suriname: Kasikasima	2.976883, -55.411385	KY679835
*T. emarginatus*	SLE482	Suriname: Kasikasima	2.976883, -55.411385	KY679836
*T. emarginatus*	SLE483	Suriname: Kasikasima	2.976883, -55.411385	KY679837
*T. kasikasima*	SLE1045	Suriname: Kappel Airstrip	3.791317, -56.149467	KY679850
*T. kasikasima*	SLE1046	Suriname: Kappel Airstrip	3.791317, -56.149467	KY679851
*T. kasikasima*	SLE1048	Suriname: Tafelberg Summit	3.926667, -56.188332	KY679849
*T. kasikasima*	SLE1049	Suriname: Tafelberg Summit	3.926667, -56.188332	KY679852
*T. kasikasima*	SLE1050	Suriname: Tafelberg Summit	3.926667, -56.188332	KY679848
*T. kusad*	SLE1021	Guyana: Kusad Mts.	2.80885, -59.865	KY679846
*T. pallidus*	SLE525	Venezuela: Tobogan de la Selva	5.386783, -67.615364	KY679853
*T. sipaliwini*	SLE422	Suriname: Kasikasima	2.976883, -55.411385	KY679838
*T. sipaliwini*	SLE478	Suriname: Kasikasima	2.976883, -55.411385	KY679839
*T. sipaliwini*	SLE497	Suriname: Kasikasima	2.976883, -55.411385	KY679840
*T. sipaliwini*	SLE1020	Guyana: Kusad Mts.	2.80885, -59.865	KY679841
*T. sipaliwini*	SLE1023	Suriname: Kwamala	2.182883, -56.787251	KY679842
*T. striatus*	SLE423	Suriname: Kasikasima	2.976883, -55.411385	KY679847
*T. sulcatus*	SLE0035	Venezuela: Tobogan de la Selva	5.386783, -67.615364	KC935327
*T. sulcatus*	SLE1027	Venezuela: Tobogan de la Selva	5.386783, -67.615364	KY679845
*T. sulcatus*	SLE1035	Venezuela: Tobogan de la Selva	5.386783, -67.615364	KY679843
*T. sulcatus*	SLE1037	Venezuela: Pijiguaos	6.593617, -66.820633	KY679844
*T.* sp. A	SLE526	Venezuela: Tobogan de la Selva	5.386783, -67.615364	KY679854

## Results

**Figure 1.**
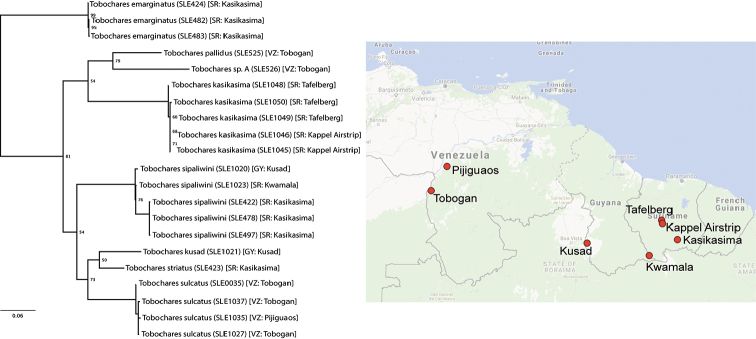
Maximum likelihood phylogeny of *Tobochares* spp. based on COI sequences, with map showing the localities of the sampled populations.

The results of the maximum likelihood analysis (Fig. 1) of the COI sequence data supported the hypothesis that the morphologically differentiated species are also genetically distinct. For species for which we sequenced multiple representatives, the amount of raw intraspecific genetic divergence among individuals of *T. emarginatus*, *T. kasikasima*, and *T. sulcatus* was less than 1%. Within individuals of *T. sipaliwini*, the maximum raw genetic divergence found was 2.5%. The raw pairwise genetic distance between any two species in the tree was greater than 10% with the exception of *T. kusad* and *T. striatus*, in which it was 7.4%. Taken together with consistent morphological differences, we found support for ten distinct species among the material we examined.

### List of species

1. *Tobochares canaliculatus* sp. n. Venezuela (Amazonas)

2. *Tobochares canthus* sp. n. Venezuela (Amazonas)

3. *Tobochares emarginatus* sp. n. Suriname

4. *Tobochares kasikasima* Short, 2013 Suriname

5. *Tobochares kusad* sp. n. Guyana

6. *Tobochares pallidus* sp. n. Venezuela (Amazonas, Bolivar)

7. *Tobochares sipaliwini* Short & Kadosoe, 2011 Suriname, Guyana

8. *Tobochares striatus* Short, 2013 Suriname

9. *Tobochares sulcatus* Short & García, 2007 Venezuela (Amazonas, Bolivar)

10. *Tobochares* sp. A Venezuela (Amazonas)

### Characters of taxonomic importance

Species of *Tobochares*, in general, are morphologically well-defined and we found relatively little variation within species.

**Figure 2.**
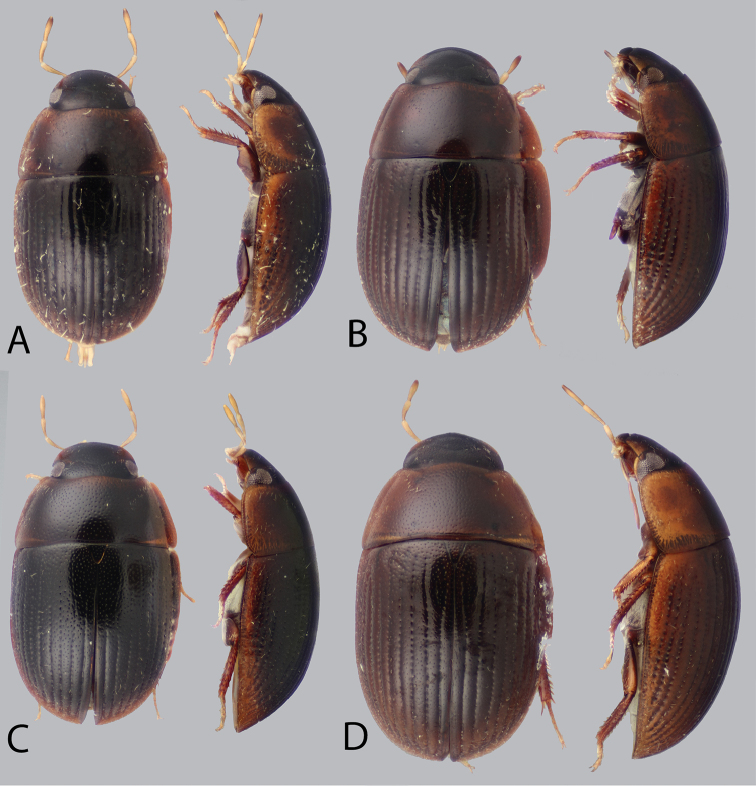
Dorsal and lateral habitus of *Tobochares* spp. **A** *T. sulcatus* **B** *T. striatus* **C** *T. sipaliwini* **D** *T. kusad* sp. n.

**Figure 3.**
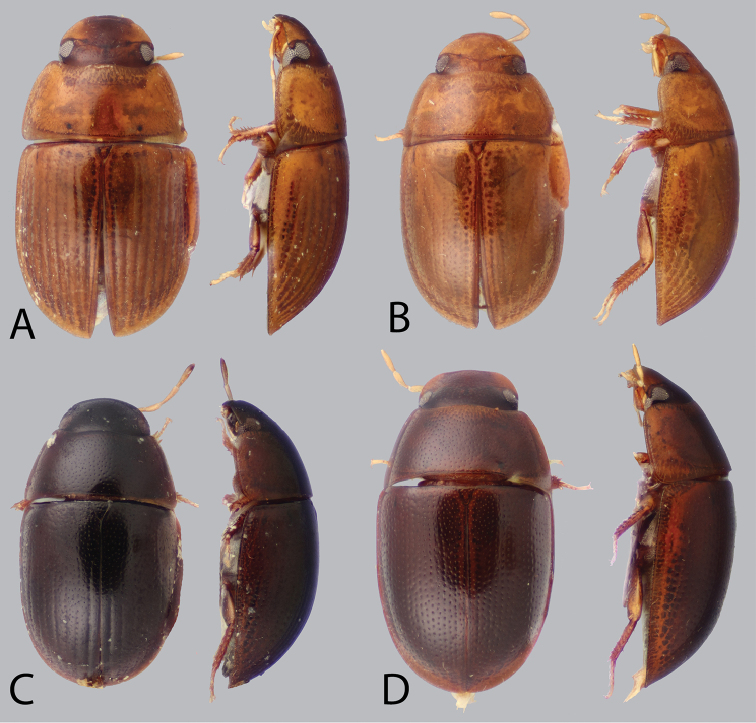
Dorsal and lateral habitus of *Tobochares* spp. **A** *T. canaliculatus* sp. n. **B** *T. pallidus* sp. n. **C** *T. kasikasima* **D** *T. canthus* sp. n.

**Dorsal coloration.** The dorsal coloration of most species is medium to dark brown (Figs 2A–D, 3C–D), but may be extremely pale, almost appearing yellow (Fig. 3A–B) in a few species. This is not due to being teneral but is the true coloration of the adult beetle (care must be taken that teneral specimens of other, darker, species are not confused with non-teneral specimens). The coloration of the head is particularly helpful in diagnosing species, as some species may have an entirely black head while others have pale preocular patches of varying sizes (e.g. Fig. 6A–F).

**Head**. A lateral canthus of the frons emarginates the eye to some extent in all *Tobochares* species (e.g. Fig. 5D), while it nearly divides the eye in two in a few species (Fig. 6D); the degree of emargination is usually consistent within species. The coloration of the apex of the maxillary palps is helpful at separating some species. In some, the entire palpomere is pale (e.g. Fig. 8D), while in others the tip is darkened (e.g. Fig. 8A–C). Like most coloration characters, there is some variation and it should not be alone used for definitive identification.

**Thoracic venter**. The condition of the mesoventrite slightly varies between species, but is not extremely useful for making identifications. In most species, it possesses a low transverse ridge which may vary in elevation (Fig. 9D–F). In a few species, the ridge is indistinct or absent, and instead represented by a bulge (Fig. 9A). The metaventrite has a distinct glabrous patch posteromedially (Fig. 10), and the size of this patch can vary between species.

**Figure 4.**
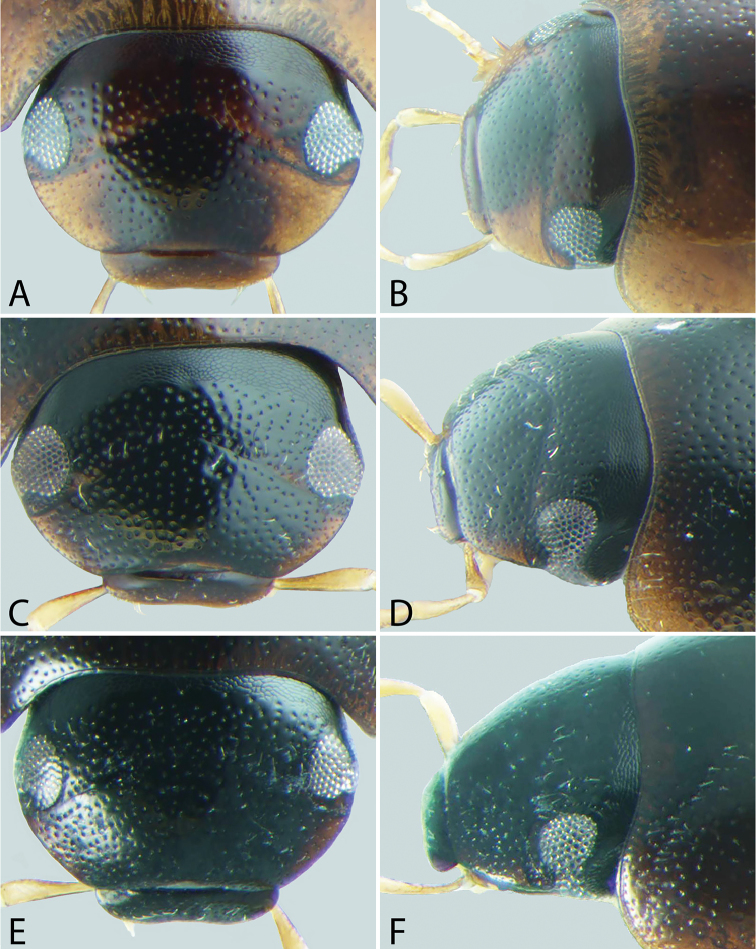
Front and lateral view of heads of *Tobochares* spp. **A–B** *T. canaliculatus* sp. n. **C–D** *T. sipaliwini* **E–F** *T. kasikasima*.

**Elytra.** The condition of the elytra is extremely useful for separating species (Fig. 11). While all species have rows of serial punctures, how much these rows are impressed into striae varies from not at all (e.g. *T. emarginatus*) to deeply along their entire length (e.g. *T. sulcatus*, Fig. 11F). The form and strength of the ground punctation also varies between species.

**Abdomen.** The surface of the abdomen is densely pubescent in all species, but some species have cuticular projections mixed in amongst the setae (Fig. 13A), while others lack these projections (Fig. 13B). The aedeagus, particularly the shape of the parameres, is diagnostic for all species.

#### Genus Tobochares

Taxon classificationAnimaliaORDOFAMILIA

Short & García, 2007

Tobochares Short & García, 2007: 2.

##### Type species.

*Tobochares sulcatus* Short & García, by original designation.

##### Differential diagnosis.

Size small, 1.5–2.4 mm. Antennae with eight antennomeres, including three-segmented club. Elytra without sutural stria, but with serial punctures impressed into distinct grooves (serial punctures present but not impressed into groves in *T. canthus*, *T. emarginatus*, and *T. pallidus*). Median elevation of mesoventrite low, forming a narrow transverse ridge or elevated bulge (Fig. 9). Metaventrite with distinct posteromedial ovoid glabrous patch (Fig. 10). Metafemora glabrous except for a few scattered setae (Fig. 12). Fifth abdominal ventrite evenly rounded, without apical emargination or coarse setae (Fig. 13). Aedeagus with basal piece very short (Fig. 14).

##### Description.

**Figure 5.**
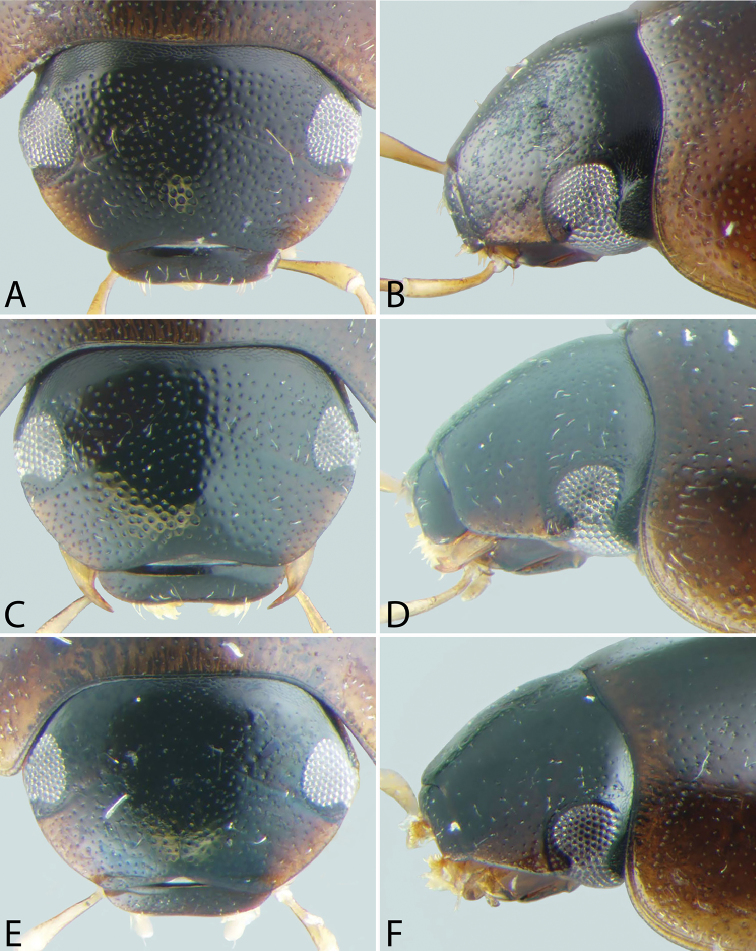
Front and lateral view of heads of *Tobochares* spp. **A–B** *T. kusad* sp. n. **C–D** *T. striatus* **E–F** *T. sulcatus*.

**Figure 6.**
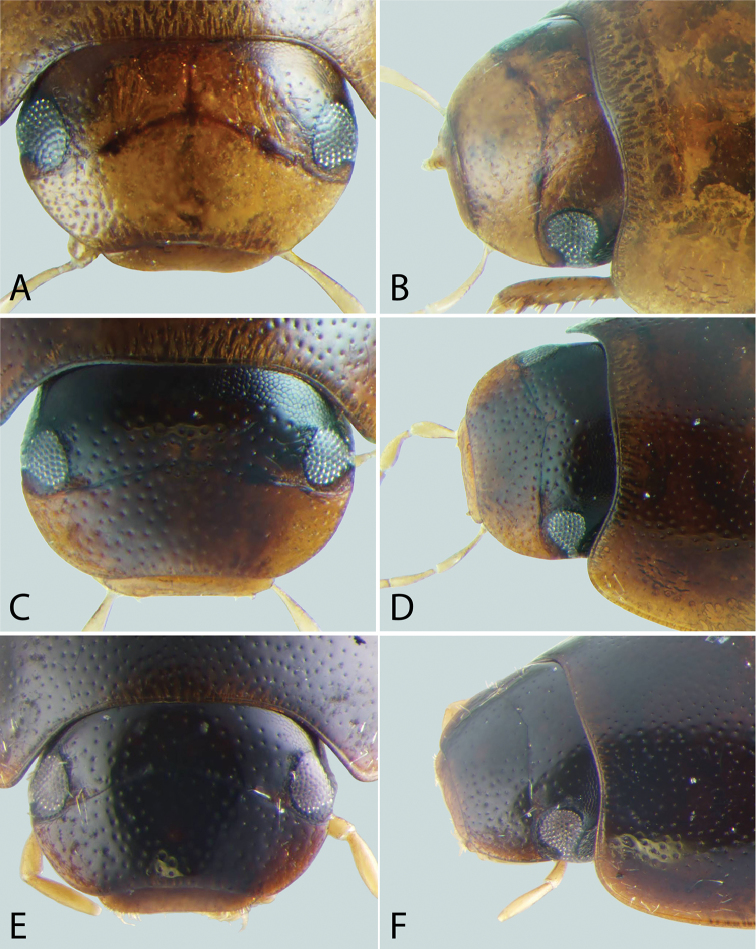
Front and lateral view of heads of *Tobochares* spp. **A–B** *T. pallidus* sp. n. **C–D** *T. canthus* sp. n. **E–F** *T. emarginatus* sp. n.

**Head.** Antennae with eight antennomeres, including three-segmented pubescent club. Maxillary palps curved inward and moderately long, as long or longer than the width of head just anterior to eyes; inner face of palpomere 2 straight to slightly curved; apical palpomere slightly longer than penultimate. Labial palps short, distinctly shorter than mentum width. Mentum flat and set with a few scattered setae; strongly emarginated anteromedially with a notch extending posteriorly about one-quarter to one-third of its length. Head with ground punctures. Frons with series of irregular systematic, setae-bearing punctures anterior to each eye. Systematic punctures also present on clypeus and labrum but blend with ground punctation. Eyes not bulging, continuous with outline of the head; slightly to strongly emarginated anteriorly by a small extension of the frons (Figs 4–6). **Thorax.** Pronotum with systematic punctation in lateral thirds, each puncture usually bearing a short seta. Prosternum narrow, not carinate medially; very slightly elevated in anterior third, and with a transverse crease. Mesoventrite with anapleural sutures distinctly concave. Mesoventrite with a low, transverse ridge medially (Fig. 9B–F) or rarely with only a weakly elevated bulge (Fig. 9A) without clear directionality (in *T. canthus* and *T. emarginatus*). Metaventrite with medium to large glabrous patch posteromedially (Fig. 10). Elytra without sutural stria; with ten rows of serial punctures which are depressed into grooves on at least the posterior half of the elytra (except *T. canthus* and *T. emarginatus*, which have weakly differentiated serial punctures that are not impressed into grooves); with irregular rows of small but distinct systematic punctures bearing short setae. Procoxae set with sparse setae, but without thickened spines. Hind femora glabrous, with only a few scattered setae (Fig. 12). All tarsi with five segments; with a few short setae on dorsal face but without long natatory setae. Ventral surface of tarsomeres 1–4 set with two rows of moderately long articulated spicules. **Abdomen.** With five densely pubescent ventrites, with setae slightly denser medially on each ventrite (Fig. 13); sometimes with small spicules interspersed amongst the setae. Fifth ventrite evenly rounded and without any thickened setae at apex. Aedeagus (Fig. 14) with short basal piece, less than one-third the length of the parameres. Median lobe moderately wide, as wide or wider than base of each paramere.

**Larvae.** The immature stages are unknown.

##### Distribution.

Venezuela (Amazonas, Bolivar), Guyana, Suriname.

##### Biology.

Nearly all collections of *Tobochares* are associated with hygropetric habitats, e.g. thin water films on rock. A handful of specimens have been collected from stream or forest pool margins that are fed by or adjacent to such habitats. Most seepages on which *Tobochares* tend to be found are littered with leaves and detritus (e.g. Figs 17A–B, 18A–B), though this is not always the case in some vertical seepage situations (e.g. Fig. 16E). The genus frequently co-occurs with other known seep specialists, including the hyrophilid genera *Oocyclus* Sharp, 1882 and *Radicitus* Short & García, 2014, the dytiscid genus *Fontidessus* Miller & Spangler, 2008, and several myxophagan groups.

#### Tobochares canaliculatus sp. n.

Taxon classificationAnimaliaORDOFAMILIA

http://zoobank.org/177BBE60-D03B-4B44-A80A-5E28F1AA46CC

Figs 3A , 4A–B , 8D , 9B , 10B , 11E , 12E , 14G , 15 , 18A–B

##### Type material.

**Holotype (male)**: “VENEZULEA: Amazonas State/ 5°23.207'N, 67°36.922'W, 125m/ Tobogan de la Selva; 8.viii.2008/ leg. A. Short, M. García, L. Joly/ AS-08-080b; old “tobogancito”/ on seepage area w/ detritus”, “[barcode]/ SEMC0877724/ KUNHM-ENT” (MIZA). **Paratypes (23): VENEZUELA: Amazonas**: same data as type (10 exs., SEMC, MALUZ; includes 1 female mounted on SEM stub); same locality but 14.i.2009, leg. Short & Miller, “partly shaded wet rock w/ algae”, VZ09-0114-01G (9 exs., SEMC); same locality but 14.i.2009, leg. Short, “clumps of wet leaves on rock”, VZ09-0114-01D (3 exs., SEMC); same locality but 23.ii.1986, P.J. Spangler, sandy margin, Colln. #10 (1 ex., USNM).

##### Differential diagnosis.

The combination of the pale dorsal coloration and deeply sulcate elytra along their entire length (Fig. 3A) will easily separate *Tobochares canaliculatus* from its congeners. The genitalia is also distinctive in having very narrow parameres which are longer than the median lobe (Fig. 14G). Only *T. pallidus* is paler in coloration, but that species lacks deeply grooved elytra. Other species with deeply sulcate elytra (e.g. *T. sulcatus*, *T. striatus*, *T. kusad*) are all very dark brown in color, and also have the tips of their parameres distinctly expanded.

##### Description.

***Size and form***: Body length 1.6–2.0 mm. Body elongate oval, moderately dorsoventrally compressed. ***Color and punctation*.** Dorsum of head brown to dark brown, anterolateral margins of clypeus with prominent pale preocular patches (Fig. 4A–B); maxillary palps distinctly pale. Pronotum light brown with the lateral margins slightly paler; elytra light brown to brown, slightly paler at lateral margins and posteriorly (Fig. 3A). Meso- and metathoracic ventrites dark brown, and abdominal ventrites very dark brown (nearly black), with prosternum slightly paler; epipleura, legs, labial palps, and antennae distinctly paler, with antennal club slightly darker than proximal antennal segments. Ground punctation on head, pronotum and elytra moderately fine. ***Head*.** Eyes measuring ~100µm anteroposteriorly, continuous with outline of head, emarginate at lateral margin, narrowing to half to slightly more than a third of the width. ***Thorax*.** Elytra with ten rows of serial punctures which are depressed into deep, smooth grooves running the full length of the elytra (Fig. 11E). Metafemora mostly glabrous on ventral face, with narrow band of pubescence along proximal third of anterior margin (Fig. 12E). Elevation of mesoventrite forming a low transverse carina, not quite elevated to the same plane as the ventral surface of the mesocoxae (Fig. 9B). Metaventrite with distinct median ovoid glabrous area that is more than half of the total metaventrite length, and about half as wide as it is long (Fig. 10B). ***Abdomen*.** Abdominal ventrites uniformly and very densely pubescent, with small spicules interspersed amongst the setae (e.g. Fig. 13A). Aedeagus (Fig. 14G) with parameres relatively narrow, nearly half as narrow as the median lobe; parallel sided in apical half and slightly convex in basal half; apex of parameres very slightly outwardly curved and about equal to the length of the median lobe; gonopore situated at the tip of the median lobe.

##### Etymology.

Dimunuative of *canalis*, referring to the elytral grooves.

##### Distribution.

Known only from the type locality in Venezuela (Fig. 15).

##### Biology.

This species has been collected on several occasions on rock seepages along the margin of the Rio Coromoto (Fig. 18A).

#### Tobochares canthus sp. n.

Taxon classificationAnimaliaORDOFAMILIA

http://zoobank.org/A2946B0F-ED87-4890-848E-7D2DF2CF41EA

Figs 3D , 6C–D , 7D , 9A , 10A , 12D , 13B , 14H , 15 , 18A–B

##### Type material.

**Holotype (male)**: “VENEZULEA: Amazonas State/ 5°23.207'N, 67°36.922'W, 125m/ Tobogan de la Selva; 8.viii.2008/ leg. A. Short, M. García, L. Joly/ AS-08-080b; old “tobogancito”/ on seepage area w/ detritus”, “[barcode]/ SEMC0877726/ KUNHM-ENT” (MIZA). **Paratypes (28): VENEZUELA: Amazonas**: same data as type (28 exs., SEMC, MALUZ, MIZA; includes 1 male mounted on SEM stub).

##### Differential diagnosis.

The lack of impressed striae on the elytra (Fig. 3D), strongly emarginated eye (Figs 6C-D), lack of spicules on the surface of the abdominal ventrites, and extremely broad glabrous patch on the metaventrite (Fig. 10A) all serve to easily separate this species from other *Tobochares* with the exception of *T. emarginatus*. It may be separated from *T. emarginatus* by the paler dorsal coloration and the shape of the aedeagus (Fig. 14H).

##### Description.

**Figure 7.**
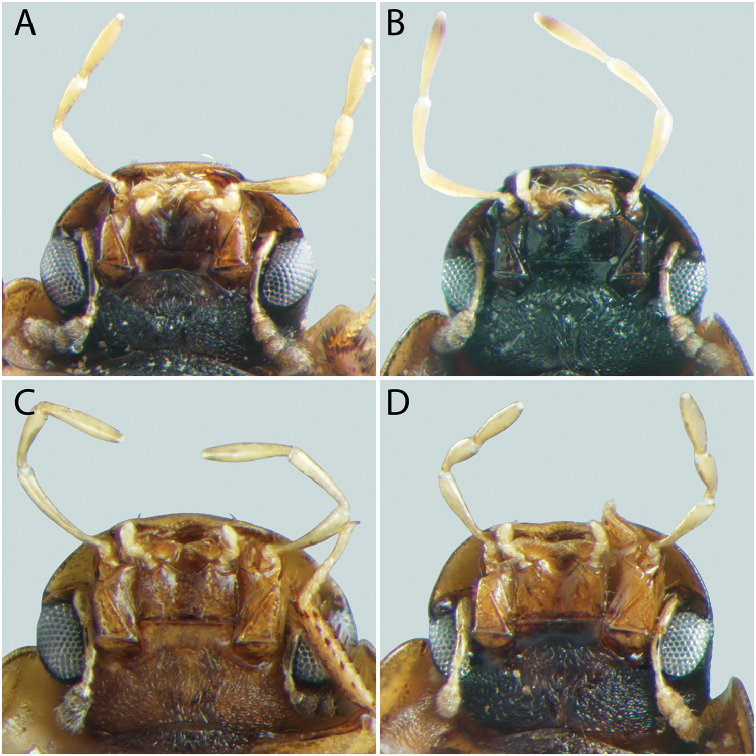
Ventral view of heads of *Tobochares* spp. **A** *T. sipaliwini* **B** *T. kasikasima* **C** *T. pallidus* sp. n. **D** *T. canthus* sp. n.

***Size and form***: Body length 1.7–2.0 mm. Body elongate oval, moderately dorsoventrally compressed. ***Color and punctation*.** Dorsum of head dark brown, frons darker (nearly black) laterally and around eyes, anterolateral margins of clypeus with paler preocular patches (Fig. 6C–D); maxillary palps distinctly pale (Fig. 7D). Pronotum dark brown with the lateral margins paler; elytra dark brown, slightly paler at lateral margins and posteriorly. Meso- and metathoracic ventrites and abdominal ventrites very dark brown, with prosternum slightly paler; legs, labial palps, and antennae distinctly paler. Ground punctation on head, pronotum and elytra moderately fine. ***Head*.** Eyes measuring ~90µm anteroposteriorly, continuous with outline of head, emarginate at lateral margin, narrowing to about a fourth of the width (Fig. 6C–D). ***Thorax*.** Elytra with punctures loosely organized into rows. Metafemora mostly glabrous on ventral face (Fig. 12D). Elevation of mesoventrite forming a low transverse carina, not quite elevated to the same plane as the ventral surface of the mesocoxae (Fig. 9A). Metaventrite with distinct median ovoid glabrous area that is more than half of the total metaventrite length, and nearly as wide as it is long (Fig. 10A). ***Abdomen*.** Abdominal ventrites uniformly and densely pubescent (Fig. 13B). Aedeagus (Fig. 14H) with parameres narrow, slightly less than half as wide as the median lobe; generally straight and parallel sided along entire length; apex of parameres not expanded, slightly tapered to a blunt tip on the medial corner; apex of median lobe distinctly surpassing the apex of the parameres; gonopore situated just below the apex of the median lobe.

##### Etymology.

Named after the condition of the eyes, in which a lateral canthus of the frons partially divides them into lower and upper surfaces.

##### Distribution.

Known only from the type locality in Venezuela (Fig. 15).

##### Biology.

This species was collected on a rock seepage along the margin of the Rio Coromoto (Fig. 18A–B). The seepage drains water from the surrounding area and is not fed directly by the creek itself. The rock surface had scattered leaves and detritus, as well as algae in some patches.

#### Tobochares emarginatus sp. n.

Taxon classificationAnimaliaORDOFAMILIA

http://zoobank.org/A623B8AD-DEC7-4F2B-8DD4-9A49639AED02

Figs 6E–F , 14I , 15 , 16C–D, F

##### Type material.

**Holotype (male)**: “SURINAME: Sipaliwini District/ N2°58.613', W55°24.683', 400m/ Camp 4 (high) Kasikasima/ leg. A. Short; main seepage area/ 24.iii.2012; SR12-0324-01C/ 2012 CI-RAP Survey”, “[barcode]/ SEMC1088469/KUNHM-ENT” (NZCS). **Paratypes (15): SURINAME: Sipaliwini**: Same data as type (11 exs., SEMC, NZCS; includes DNA vouchers SLE424, SLE482, and SLE483); Camp 1, Upper Palumeu, 11.iii.2012, leg. A. Short, around waterfall, SR12-0311-03A (1 ex., SEMC); Raleighvallen Nature Reserve, Voltzberg trail, 30.vii.2012, leg. Short & McIntosh, detrital pools along stream, SR12-0730-01B (2 exs., SEMC).

##### Differential diagnosis.

The lack of impressed striae on the elytra, strongly emarginated eye (Fig. 6E–F), lack of spicules on the surface of the abdominal ventrites, and extremely broad glabrous patch on the metaventrite (e.g. Fig. 10A) all serve to easily separate this species from other *Tobochares* with the exception of *T. canthus*. It may be separated from *T. canthus* by the darker dorsal coloration and the shape of the aedeagus (Fig. 14I).

##### Description.

***Size and form***: Body length 1.7–2.1 mm. Body elongate oval, moderately dorsoventrally compressed. ***Color and punctation*.** Dorsum of head brown, frons darker laterally and around eyes, anterolateral margins of clypeus with paler preocular patches (Fig. 6E–F); maxillary palps distinctly pale. Pronotum brown with the lateral margins paler; elytra brown, slightly paler at lateral margins and posteriorly. Meso- and metathoracic ventrites and abdominal ventrites brown to dark brown, with prosternum slightly paler; legs, labial palps, and antennae distinctly paler. Ground punctation on head, pronotum and elytra moderately fine. ***Head*.** Eyes measuring ~90µm anteroposteriorly, continuous with outline of head, emarginate at lateral margin, narrowing to about a fourth of the width (Fig. 6E–F). ***Thorax*.** Elytra with punctures loosely organized into rows. Metafemora mostly glabrous on ventral face. Elevation of mesoventrite forming a low transverse carina, not quite elevated to the same plane as the ventral surface of the mesocoxae. Metaventrite with distinct median ovoid glabrous area that is more than half of the total metaventrite length, and nearly as wide as it is long. ***Abdomen*.** Abdominal ventrites uniformly and densely pubescent. Aedeagus (Fig. 14I) with parameres moderately narrow, less than half as wide as the median lobe; straight and parallel sided in basal two-thirds, then bending inward and tapering on apical third; apex of parameres nearly flat, not expanded, tapered to a blunt tip on the medial corner; apex of median lobe slightly surpassing the apex of the parameres; gonopore situated just below the apex of the median lobe.

##### Etymology.

Named after the condition of the eyes, in which a lateral canthus of the frons partially divides them into lower and upper surfaces.

##### Distribution.

Known from several localities in central and southern Suriname (Fig. 15).

##### Biology.

The majority of specimens were collected on a large granite seepage on Kasikasima (Fig. 16F), while one specimen was taken by floating clumps of leaves that were on wet rocks next to a large cascade (Fig. 16C–D) in a bucket of water. One specimen was taken from detrital pools along a stream.

#### Tobochares kasikasima

Taxon classificationAnimaliaORDOFAMILIA

Short, 2013

Figs 3C , 4E–F , 7B , 9D , 10D , 11B , 12A , 13A , 14E , 15 , 16F , 17C–F

Tobochares kasikasima Short, 2013: 83.

##### Type material examined.

**Holotype** (male): “SURINAME: Sipaliwini District/ N2°58.613', W55°24.683', 400m/ Camp 4 (high) Kasikasima/ leg. A. Short; main seepage area/ 24.iii.2012; SR12-0324-01C/ 2012 CI-RAP Survey”, “[barcode]/ SEMC1088588/KUNHM-ENT” (NZCS).

##### Additional material examined

**(59). SURINAME: Sipaliwini**: Same data as type (1 ex., SEMC); Central Suriname Nature Reserve, near Kappel airstrip, leg. Short & Bloom, 24.viii.2013, seepage flowing into canal/ditch on S. side of airstrip, SR13-0824-02B (32 exs., SEMC, NZCS, includes DNA vouchers SLE1045, SLE1046); Same data but canal/ditch on S. side of airstrip, SR13-0824-02A (1 ex., SEMC); Central Suriname Nature Reserve, Tafelberg Summit, nr. Augustus Creek Camp, large seepage area, leg. Short & Bloom, 14.viii.2013, large seepage area, SR13-0814-03A (1 ex., SEMC); Central Suriname Nature Reserve, Tafelberg Summit, nr. Caiman Creek Camp, leg. Short & Bloom, 19.viii.2013, large seepage area, SR13-0819-01A (13 exs., SEMC, includes DNA vouchers SLE1048, SLE1049); same locality but leg. Short & Bloom, 20.viii.2013, washing seepage, SR13-0820-05A (11 exs., SEMC, including 2 specimens mounted on SEM stubs and DNA voucher SLE1050).

##### Differential diagnosis.

The weakly impressed striae limited to the posterior third of the elytra (Fig. 11B), darkened apex of the maxillary palps (Fig. 7B), and overall darker body coloration serve to distinguish this species. The aedeagus of *T. kasikasima* (Fig. 14E) is also one of the most distinct within the genus, with its extremely long narrow parameres and broad, parallel sided basal median lobe. It is most similar to *T. sipaliwini* which has more extensive elytral grooves, pale maxillary palps, and an aedeagus of a much different form.

##### Description.

***Size and form*.** Body length 1.6–2.0 mm. Body elongate oval, moderately dorsoventrally compressed. ***Color and punctation*.** Dorsum of head very dark brown to black, anterolateral margins of clypeus with very faint paler preocular patches (Fig. 4E–F); maxillary palps distinctly pale, apex of maxillary palpomere 4 darkened (Fig. 7B). Pronotum very dark brown with the lateral margins paler; elytra very dark brown, slightly paler at lateral margins and posteriorly (Fig. 3C). Meso- and metathoracic ventrites and abdominal ventrites very dark brown (nearly black), with prosternum slightly paler; epipleura, legs, labial palps, and antennae distinctly paler, with antennal club slightly darker than proximal antennal segments. Ground punctation on head, pronotum and elytra moderately fine. ***Head*.** Eyes measuring ~100µm anteroposteriorly, continuous with outline of head, emarginate at lateral margin, narrowing to half the width (Fig. 4E–F). ***Thorax*.** Elytra with ten rows of serial punctures which are depressed into very shallow grooves in the posterior third, with depth of the grooves greatest near the elytral suture (Fig. 11B). Metafemora mostly glabrous on ventral face, with narrow band of pubescence along proximal half of anterior margin (Fig. 12A). Elevation of mesoventrite forming a transverse carina with a faint tooth, elevated to the same plane as the ventral surface of the mesocoxae (Fig. 9D). Metaventrite with distinct median ovoid glabrous area that is approximately half to slightly more than half of the total metaventrite length (Fig. 10D), and about half as wide as it is long. ***Abdomen*.** Abdominal ventrites uniformly and very densely pubescent, with small spicules interspersed amongst the setae. Aedeagus (Fig. 14E) with parameres thin and strap-like, about as wide as the median lobe in basal half, then gradually tapering in apical half, the apex less than half the width of the median lobe; apex of parameres blunt and rounded; apex of median lobe distinctly surpassing the apex of the parameres; median lobe with margins straight and parallel sided throughout, except at apex which is bluntly rounded; gonopore apparently absent (not observed in several examined specimens).

##### Distribution.

The species was originally described from a single male from Mt. Kasikasima in south-central Suriname. It has subsequently been collected in longer series at several other localities in central and southern Suriname including from the summit of Tafelberg Tepui (Fig. 15).

##### Biology.

The first specimen of this species was found in a seepage at the base of Kasikasima (Fig. 16F). This species has been collected in long series in seepages on the summit of Tafelberg Tepui, the only table mountain in Suriname (Fig. 17E–F); these records represent the highest known collecting event for any *Tobochares* species (c. 733 m). It was also collected on a seepage along the margin of Kappel Airstrip, which is near the base of Tafelberg (Fig. 17C–D). Both Tafelberg and Kappel Airstrip seepages were on sandstone and sedimentary rock and had an abundance of algal and moss growth.

#### Tobochares kusad sp. n.

Taxon classificationAnimaliaORDOFAMILIA

http://zoobank.org/7A205A96-926F-4B27-9E4F-D0F45A3E52EF

**Figure 8.**
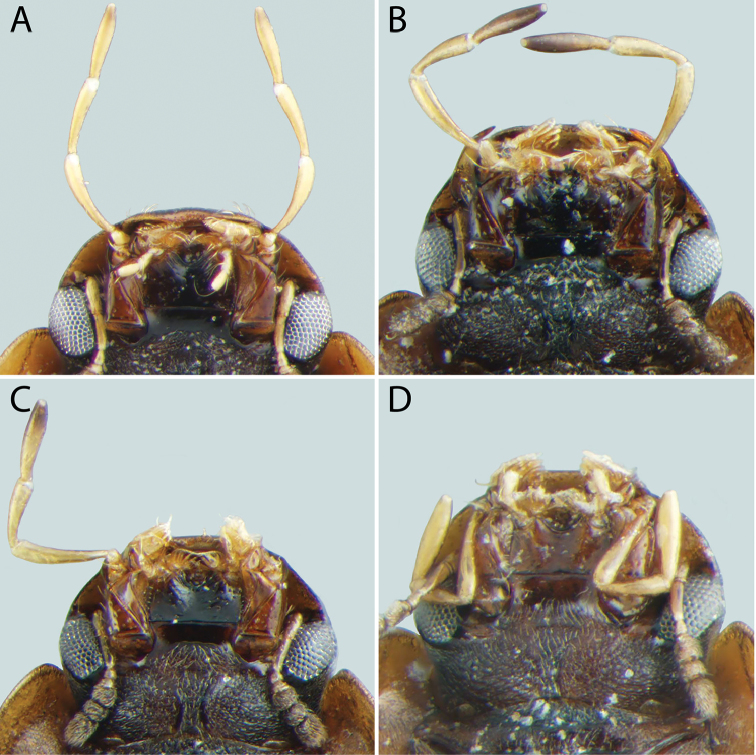
Ventral view of heads of *Tobochares* spp. **A** *T. kusad* sp. n. **B** *T. striatus* **C** *T. sulcatus* **D** *T. canaliculatus* sp. n.

Figs 2D , 5A–B , 8A , 14A , 15 , 17A–B

##### Type material.

**Holotype (male)**: “GUYANA: Region IX/ 2 48.531'N, 59 51.900'W, 170m/ Kusad Mts., Mokoro Creek/ main seepage area/ leg. Short, Isaacs, Salisbury/ 27.x.2013; GY13-1027-03B”, “[barcode]/ SEMC1271353/ KUNHM-ENT” (CBDG). **Paratypes (29): GUYANA: Region IX**: Same data as type (4 exs., SEMC); same locality but leg. Short & Washington, 24.x.2013, GY13-1024-03C (13 exs., SEMC, CBDG, NZCS; includes DNA voucher SLE1021); same locality but “small rock pool with detritus”, leg. Salisbury, 24.x.2013, GY13-1024-03A (12 exs., SEMC, CBDG).

##### Differential diagnosis.

This species can be distinguished from most species by the impressed striae running the full length of the elytra and its overall dark brown coloration (Fig. 2D). It is most similar to *T. striatus* from which it can be separated by the apex of the last maxillary palpomere only slightly darkened (Fig. 8A) at the tip (more extensive darkening in *T. striatus*; Fig. 8B) and the eyes being slightly less emarginated laterally (compare Fig. 5B, D).

##### Description.

***Size and form***: Body length 2.0–2.4 mm. Body elongate oval, moderately dorsoventrally compressed. ***Color and punctation*.** Dorsum of head very dark brown to black, anterolateral margins of clypeus with paler preocular patches (Fig. 5A–B); maxillary palps distinctly pale, with the apex of palpomere 4 slightly to significantly darker (Fig. 8A). Pronotum brown to dark brown with the lateral margins appearing slightly paler; elytra brown to very dark brown, slightly paler at lateral margins and posteriorly (Fig. 2D). Meso- and metathoracic ventrites and abdominal ventrites very dark brown (nearly black), with prosternum slightly paler; epipleura, legs, labial palps, and antennae distinctly paler, with antennal club slightly darker than proximal antennal segments. Ground punctation on head, pronotum and elytra moderately fine. ***Head*.** Eyes measuring ~150µm anteroposteriorly, continuous with outline of head, emarginate at lateral margin, narrowing to roughly two thirds of the width (Fig. 5A–B). ***Thorax*.** Elytra with ten rows of serial punctures which are depressed into grooves running the full length of the elytra. Metafemora mostly glabrous on ventral face, with narrow band of pubescence along proximal third of anterior margin. Elevation of mesoventrite forming a low transverse carina with a prominent “tooth,” elevated to the same plane as the ventral surface of the mesocoxae. Metaventrite with distinct median ovoid glabrous area that is more than half of the total metaventrite length, and about half as wide as it is long. ***Abdomen*.** Abdominal ventrites uniformly and very densely pubescent, with small spicules interspersed amongst the setae. Aedeagus (Fig. 14A) with parameres about as wide as median lobe basally, parallel sided in basal half, then gradually narrowing in apical third; apex of parameres weakly expanded and bluntly rounded. Median lobe gradually tapering to a bluntly rounded apex, which slightly extends beyond the apex of the parameres; gonopore situated distinctly below the apex of the median lobe.

##### Etymology.

Named after Kusad Mountain in the South Rupununi region of Guyana, from where the species is known.

##### Distribution.

Only known from the type locality in Guyana (Fig. 15).

##### Biology.

The species was collected on a thin rock seepage flowing over granite that was associated with a small creek (Fig. 17A–B). The seepage was mostly covered with dead leaves and detritus. Some specimens were also found in small pools in the rock that accumulated water from the seep as well as along the margins of the stream pool into which the seep flowed.

#### Tobochares pallidus sp. n.

Taxon classificationAnimaliaORDOFAMILIA

http://zoobank.org/A4BC74D6-1D77-4EDE-82DF-E988FBA208F2

Figs 3B , 6A–B , 7C , 9C , 10C , 11D , 12C , 14F , 15 , 18A–B, D–F

##### Type material.

**Holotype (male)**: “VENEZULEA: Amazonas State/ 5°23.207'N, 67°36.922'W, 125m/ Tobogan de la Selva; 8.viii.2008/ leg. A. Short, M. García, L. Joly/ AS-08-080b; old “tobogancito”/ on seepage area w/ detritus”, “[barcode]/ SEMC0877702/ KUNHM-ENT” (MIZA). **Paratypes (28): VENEZUELA: Amazonas**: same data as type (10 exs., SEMC, MALUZ, MIZA); same locality but leg. M. Balke (1 ex., SEMC; DNA voucher SLE525); same locality but 23.ii.1986, leg. Spangler, sandy margin, Colln. #10 (1 ex., USNM); same locality but 18.i.1989, leg. Spangler, Faitoute, & Barr, upper seep (1 ex., USNM); nr. Hotel Nacamtur, 5°36'16.18"N, 67°34'56.24"W, 14.ix.2007, isolated rock seep w/ algae, leg. A. Short, AS-07-013x (1 ex., SEMC). **Bolivar**: Los Pijiguaos, 6°35.617'N, 66°49.238'W, 80 m, 16.ix.2007, leg. Short, García, & Joly, morichal/rock outcrop, AS-07-015 (5 exs., SEMC); same locality but 8.vii.2010, leg Short, Tellez, Arias, small stream on outcrop, VZ10-0708-01B (4 exs., SEMC; includes one ex. mounted on SEM stub); same locality but 9.vii.2010, leg. Short, Tellez, Arias, seeps and stream at night, VZ10-0709-03A (4 exs., SEMC); ca. 15 km NE Pijiguaos, 6°57.904'N, 66°36.392'W, 51 m, 9.vii.2010, leg. Short & Tellez, rock outcrop, detritus flotation, VZ10-0709-01B (1 ex., SEMC).

**Figure 9.**
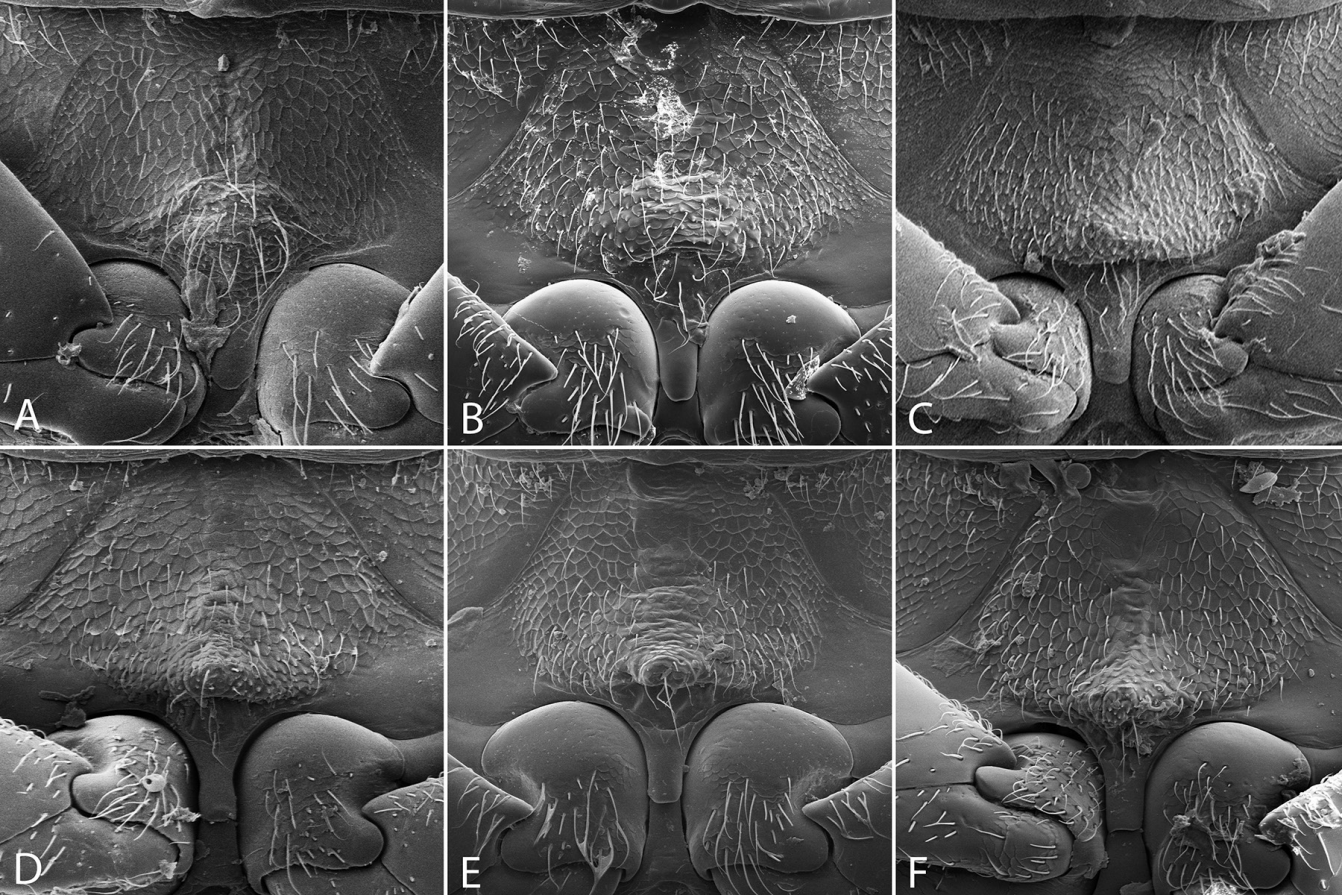
Mesoventrite of *Tobochares* spp. **A** *T. canthus* sp. n. **B** *T. canaliculatus* sp. n. **C** *T. pallidus* sp. n. **D** *T. kasikasima* **E** *T. sipaliwini* **F** *T. striatus*.

##### Differential diagnosis.

This small species may be easily distinguished by the combination of its very pale dorsal coloration and lack of impressed elytral striae (Fig. 3B). The aedeagus is also unique among *Tobochares* for its outwardly bent parameres (Fig. 14F). Only *T. canaliculatus* is also nearly as pale, but the elytra of that species are deeply grooved. *Tobochares canthus* is not typically as pale as *T. pallidus*, and *T. canthus* can be further distinguished by its deeply emarginated eyes.

##### Description.

**Figure 10.**
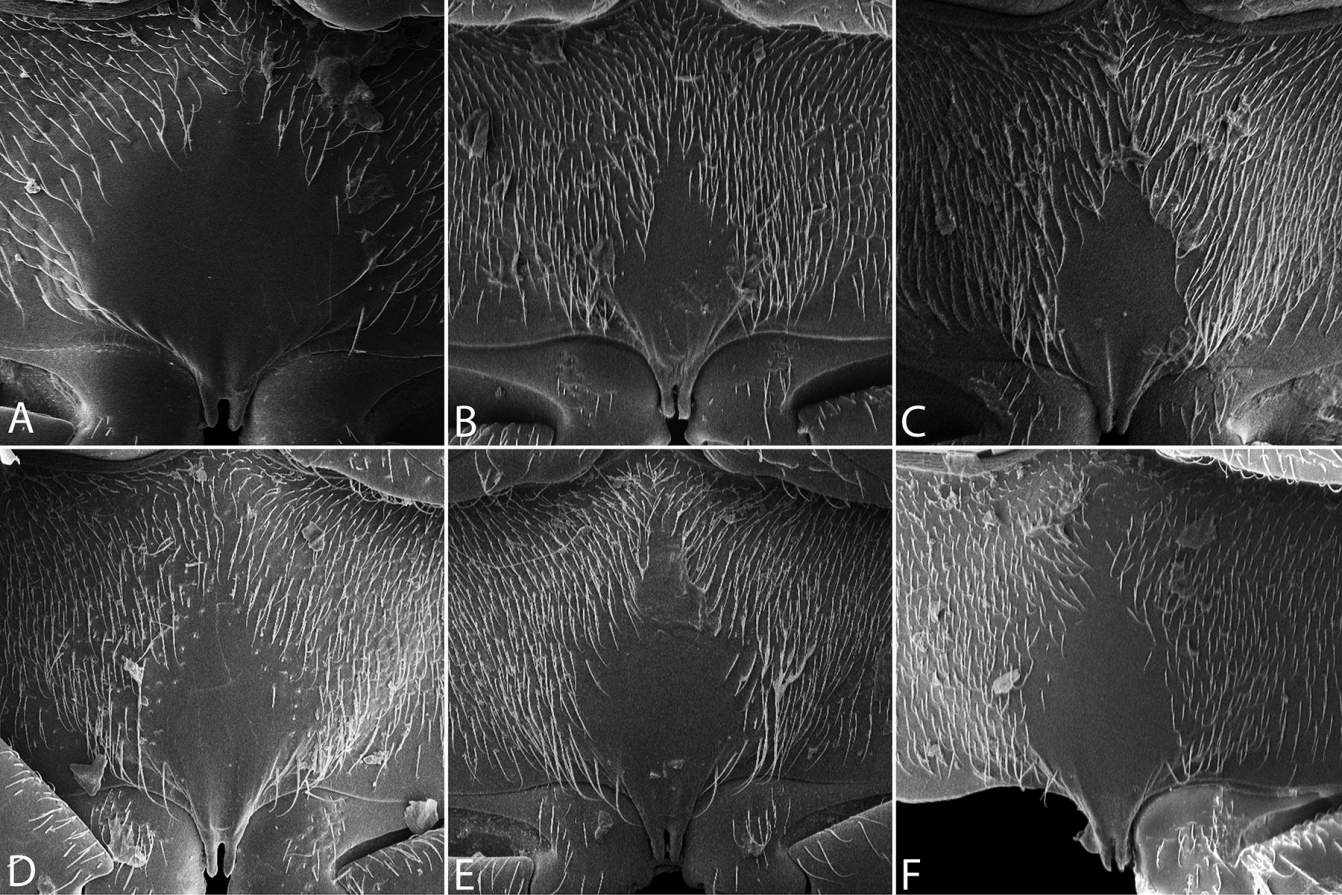
Metaventrite of *Tobochares* spp. **A** *T. canthus* sp. n. **B** *T. canaliculatus* sp. n. **C** *T. pallidus* sp. n. **D** *T. kasikasima* **E** *T. sipaliwini* **F** *T. striatus*.

**Figure 11.**
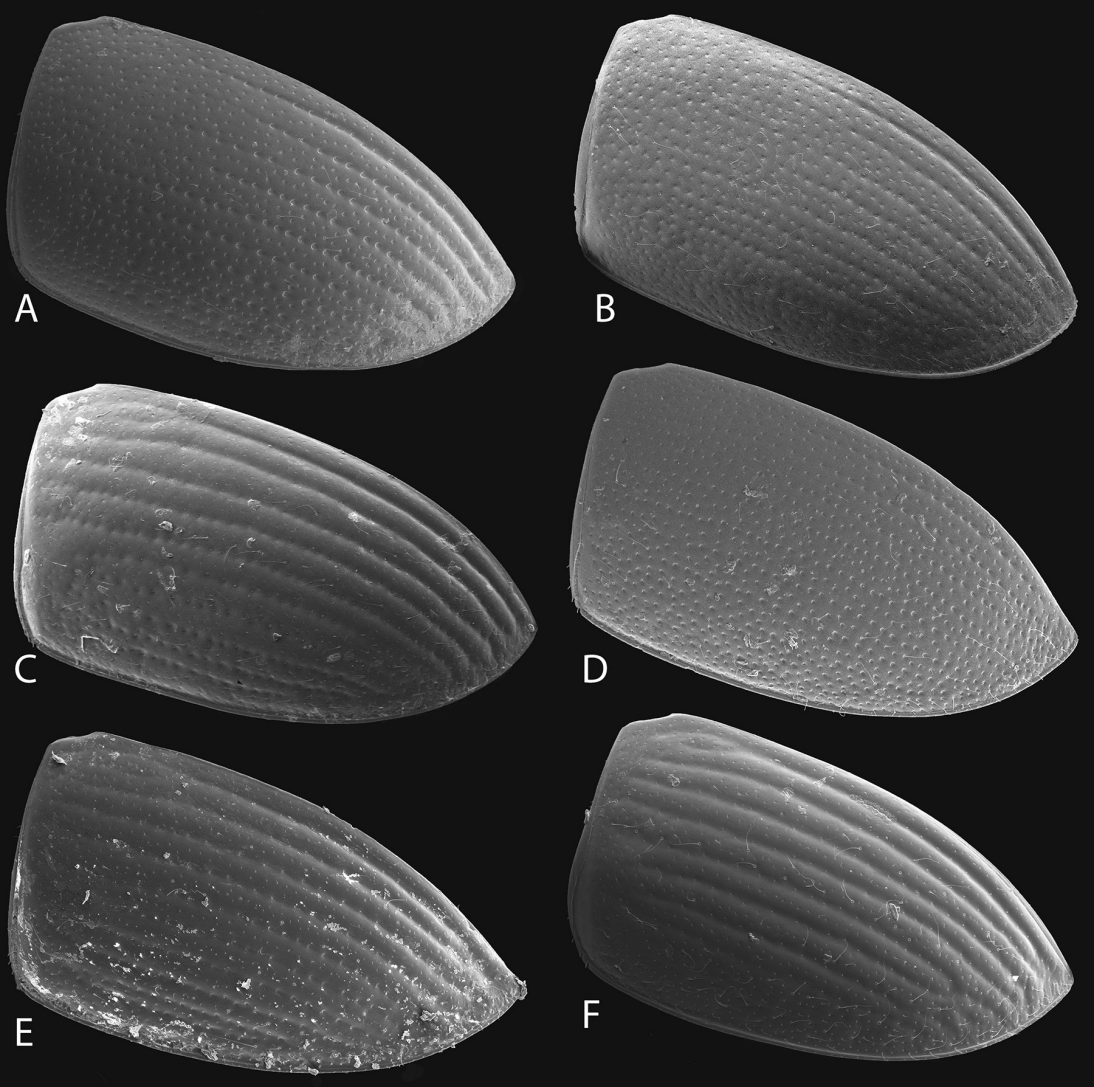
Elytra of *Tobochares* spp. **A** *T. sipaliwini* **B** *T. kasikasima* **C** *T. striatus* **D** *T. pallidus* sp. n. **E** *T. canaliculatus* sp. n. **F** *T. sulcatus*.

**Figure 12.**
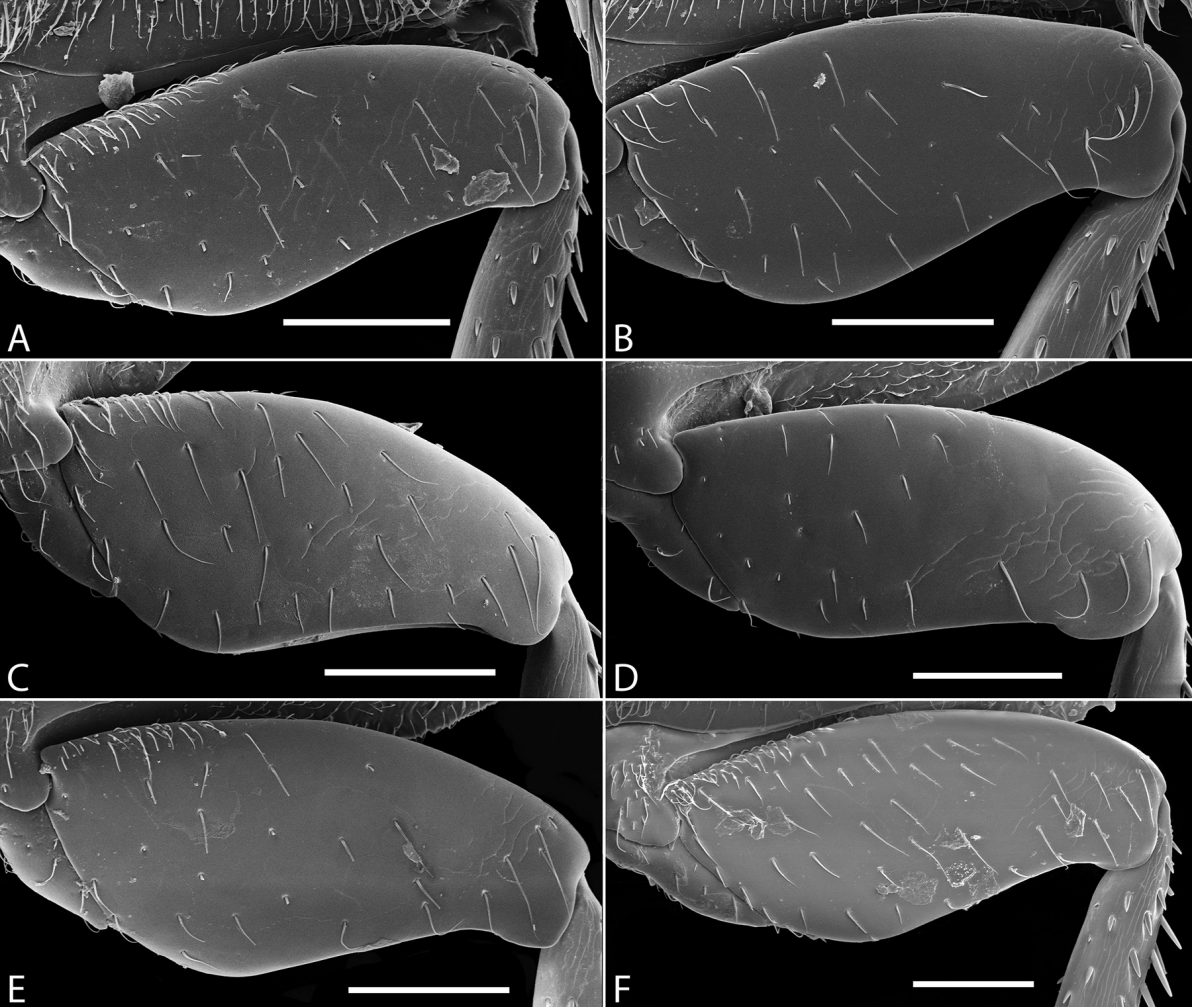
Metafemora (ventral view) of *Tobochares* spp. **A** *T. kasikasima* **B** *T. sipaliwini* **C** *T. pallidus* sp. n. **D** *T. canthus* sp. n. **E** *T. canaliculatus* sp. n. **F** *T. striatus*. Scale bars = 0.1 mm.

**Figure 13.**
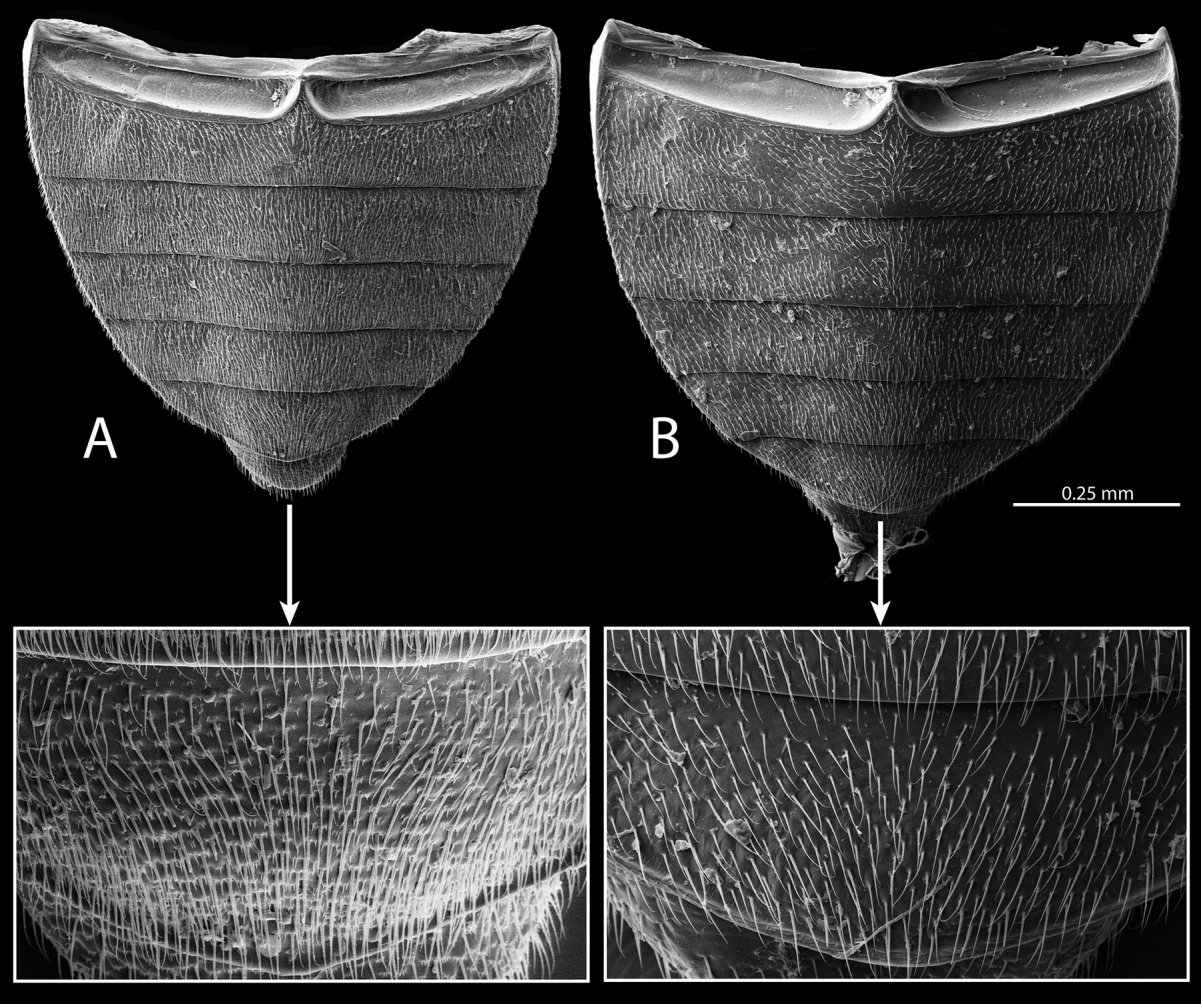
Abdominal ventrites of *Tobochares* spp. **A** *T. kasikasima* **B** *T. canthus* sp. n.

**Figure 14.**
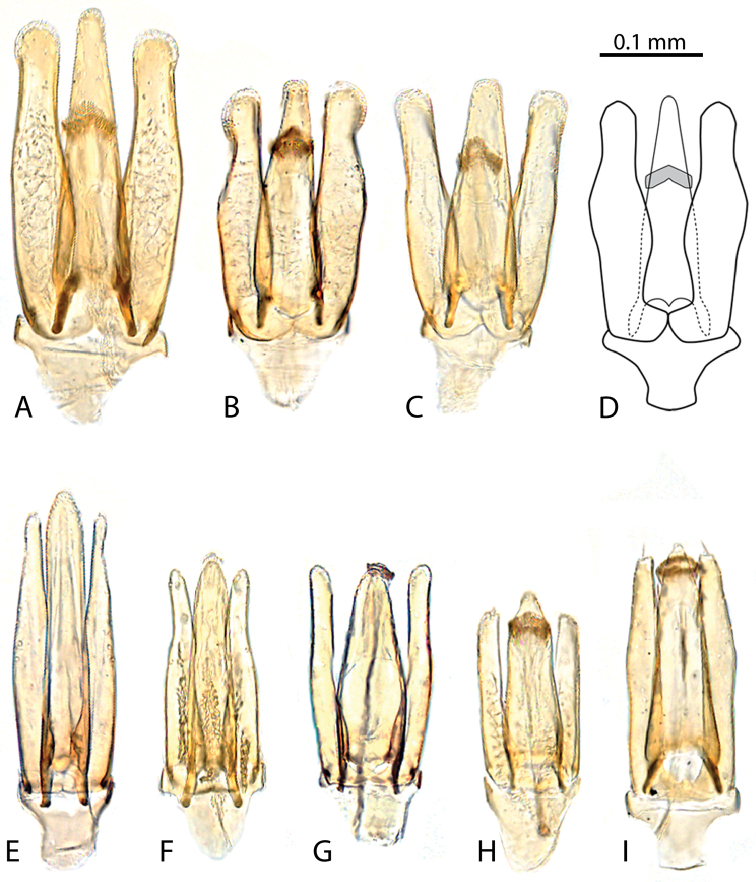
Aedeagi (ventral view) of *Tobochares* spp. **A** *T. kusad* sp. n. **B** *T. sulcatus* **C** *T. sipaliwini* **D** *T. striatus* **E** *T. kasikasima* **F** *T. pallidus* sp. n. **G** *T. canaliculatus* sp. n. **H** *T. canthus* sp. n. **I** *T. emarginatus* sp. n.

***Size and form***: Body length 1.5–1.9 mm. Body elongate oval, moderately dorsoventrally compressed. ***Color and punctation*.** Dorsum of head very pale brown; maxillary palps pale (Fig. 7C). Pronotum very pale brown; elytra very pale brown and somewhat transparent. Meso- and metathoracic ventrites brown, and abdominal ventrites very dark brown (nearly black), with prosternum distinctly paler; epipleura, legs, labial palps, and antennae pale. Ground punctation on head, pronotum and elytra moderately fine. ***Head*.** Eyes measuring ~100µm anteroposteriorly, continuous with outline of head, emarginate at lateral margin, narrowing to roughly half the width (Fig. 6A–B). ***Thorax*.** Elytra with ten rows of serial punctures, not impressed into groves (Fig. 11D). Metafemora mostly glabrous on ventral face, with narrow band of pubescence along proximal third of anterior margin (Fig. 12C). Elevation of mesoventrite forming a low transverse carina, not quite elevated to the same plane as the ventral surface of the mesocoxae (Fig. 9C). Metaventrite with distinct median ovoid glabrous area that is half to slightly more than half of the total metaventrite length, and about half as wide as it is long (Fig. 10C). ***Abdomen*.** Abdominal ventrites uniformly and very densely pubescent, with small spicules interspersed amongst the setae (e.g. Fig. 13A). Aedeagus (Fig. 14F) with parameres relatively narrow, nearly half as narrow as the median lobe; parallel sided in a little more than basal half, then narrowing and bent outwards in apical third; apex of parameres bluntly rounded and about equal to the length of the median lobe; gonopore situated at the tip of the median lobe.

##### Etymology.

Named for the species’ relatively pale body coloration.

##### Distribution.

**Figure 15.**
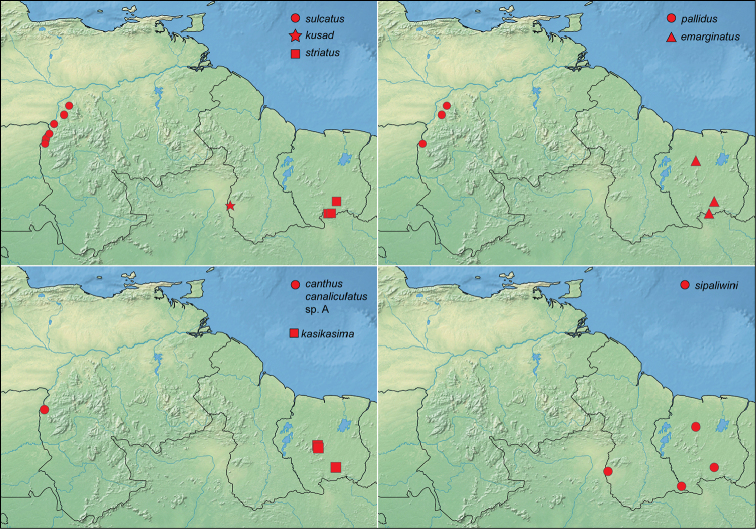
Distribution of *Tobochares* spp.

Known from several localities along the northwestern shoulder of the Guiana Shield in Venezuela (Fig. 15).

##### Biology.

All six collecting events of this species were from rock seepages. The type locality and longest series of specimens were collected on a rock seepage along the margin of the Rio Coromoto (Fig. 18A–B). The seepage drains water from the surrounding area and is not fed directly by the creek itself. The rock surface had scattered leaves and detritus, as well as algae in some patches. Other collections were on more isolated and seasonal seeps that are only flowing in the wet season (Fig. 18D).

##### Remarks.

Because this species co-occurs with several other *Tobochares* in Venezuela, the extremely pale coloration makes specimens easily confused for teneral individuals of other species in the field until they can be examined under higher magnification.

#### Tobochares sipaliwini

Taxon classificationAnimaliaORDOFAMILIA

Short & Kadosoe, 2011

Figs 2C , 4C–D , 7A , 9E , 10E , 11A , 12B , 14C , 15 , 16A–B, E–F

Tobochares sipaliwini Short & Kadosoe, 2011: 85.

##### Type material examined.

**Holotype** (male): “SURINAME: Sipaliwini District/ 2°10.973'N, 56°47.235'W, 210 m/ Camp 2, on Sipaliwini River/ leg. Short & Kadosoe; Inselberg/ 29–30. viii.2010; SR10-0829-01A/ 2010 CI-RAP Survey” (NZCS). **Paratypes (4): SURINAME: Sipaliwini District**: Same data as type (3 exs., SEMC, USNM, NZCS). Same camp but 31.viii.2010, sandy forest creek, SR10-0831-01B (1 ex., SEMC).

##### Additional material examined

**(120). SURINAME: Sipaliwini**: Same data as type except 1.ix.2010, seep on inselberg, SR10-0901-01A (1 ex., SEMC; DNA voucher SLE1023); Raleighvallen Nature Reserve, plateau below Voltzberg, 28.vii.2012, leg. Short, Maier, & McIntosh, seep/wet rocks in shaded part of trail, SR12-0728-01J (1 ex., SEMC); same data but rock pool, SR12-0728-01F (1 ex., SEMC); Raleighvallen Nature Reserve, Voltzberg trail, 30.vii.2012, leg. Short & McIntosh, detrital pools along stream, SR12-0730-01B (6 exs., SEMC); same data but leg. Maier & Kadosoe, margin of stream, SR12-0730-01A (3 exs., SEMC; includes one specimen on SEM stub); Raleighvallen Nature Reserve, base of Voltzberg, 16.iii.2016, leg. A. Short, seepage on top of rock, SR16-0316-01C (38 exs., SEMC); same data but seepage spot on side of rock, SR16-0316-01A (45 exs., SEMC); same locality but 17.iii.2016, leg. Short & Girón, flotation of roots and debris from seepage, SR16-0317-01C (5 exs., SEMC); Camp 4 (high), Kasikasima, 24.iii.2012, leg. A. Short, main seepage area, SR12-0324-01C (9 exs., SEMC; includes DNA voucher SLE422); same data but “white rock seepage area on trail”, SR12-0324-01B (2 exs., SEMC; DNA vouchers SLE478 and SLE497). **GUYANA: Region IX**: Kusad Mts., large seepage near basecamp, 24.x.2013, leg. Short & Washington, on wet rocks, GY13-1024-03C (6 exs., SEMC; includes DNA voucher SLE1020); Kusad Mts., nr. Basecamp, 24.x.2013, leg. Salisbury, small rock pool with detritus, GY13-1024-03A (3 exs., SEMC).

##### Differential diagnosis.

This species can be distinguished from most other *Tobochares* by the elytral striae being impressed only on the posterior half (Fig. 11A), and its dark brown coloration (Fig. 2C). It is most similar to *T. kasikasima* from which it may be distinguished by the more extensive elytral striae (only in posterior third in *T. kasikasima*), the uniformly pale maxillary palps (Fig. 7A), and its differently shaped aedeagus (Fig. 14C).

##### Description.

***Size and form***: Body length 1.7–2.1 mm. Body elongate oval, moderately dorsoventrally compressed. ***Color and punctation*.** Dorsum of head very dark brown to black, anterolateral margins of clypeus with paler preocular patches (Fig. 4C–D); maxillary palps distinctly pale (Fig. 7A). Pronotum very dark brown with the lateral margins paler; elytra very dark brown, slightly paler at lateral margins and posteriorly (Fig. 2C). Meso- and metathoracic ventrites and abdominal ventrites very dark brown (nearly black), with prosternum slightly paler; epipleura, legs, labial palps, and antennae distinctly paler, with antennal club slightly darker than proximal antennal segments. Ground punctation on head, pronotum and elytra moderately fine. ***Head*.** Eyes measuring ~100µm anteroposteriorly, continuous with outline of head, emarginate at lateral margin, narrowing to about half to slightly greater than half the width. ***Thorax*.** Elytra with ten rows of serial punctures which are depressed into shallow grooves in the posterior half, with depth of the grooves greatest near the elytral suture (Fig. 11A). Metafemora mostly glabrous on ventral face, with narrow band of pubescence along proximal third of anterior margin (Fig. 12B). Elevation of mesoventrite forming a low transverse carina with an acute “tooth,” elevated to the same plane as the ventral surface of the mesocoxae (Fig. 9E). Metaventrite with distinct median ovoid glabrous area that is more than half of the total metaventrite length, and about half as wide as it is long (Fig. 10E). ***Abdomen*.** Abdominal ventrites uniformly and very densely pubescent, with small spicules interspersed amongst the setae (e.g. Fig. 13A). Aedeagus (Fig. 14C) with parameres slightly wider than median lobe; parallel sided in basal half, then slightly narrowing in apical half to third; apex of parameres very slightly expanded and bluntly rounded. Median lobe gradually tapering to a bluntly rounded apex, which slightly extends beyond the apex of the parameres; gonopore situated distinctly below the apex of the median lobe.

##### Distribution.

Originally described from an inselberg on the Suriname-Guyana boarder, this species has now been found at other localities in both countries (Fig. 15).

##### Biology.

**Figure 16.**
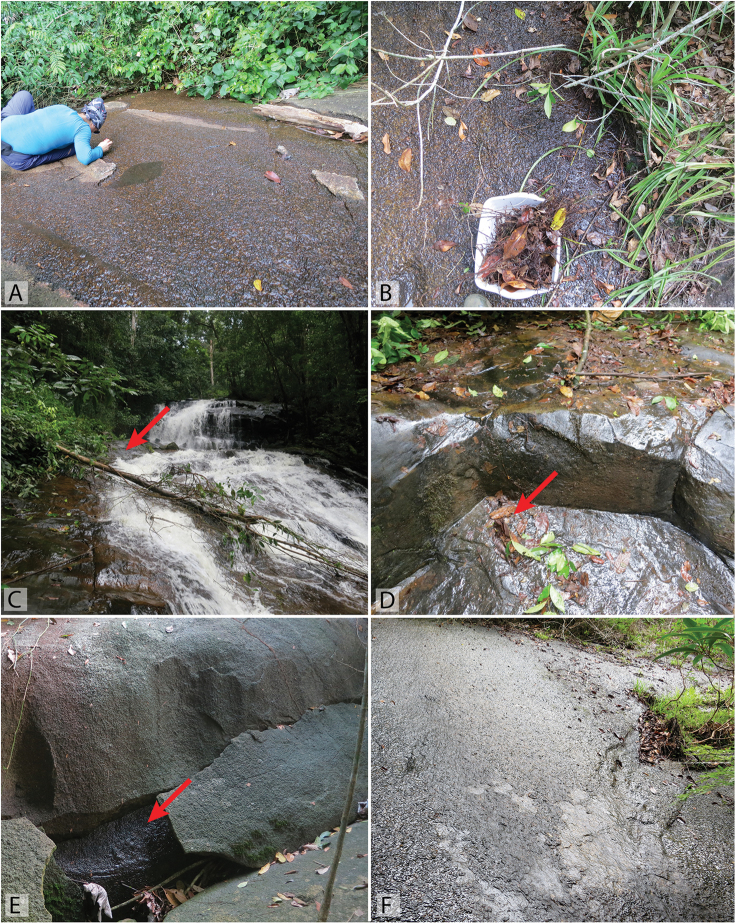
Habitat of *Tobochares* spp. in Suriname. **A–B** Base of Voltzberg, collecting event SR16-0316-01C, habitat of *T. sipaliwini* **C–D** Waterfall in the upper Palumeu watershed, collecting event SR12-0311-03A, habitat of *T. emarginatus* sp. n. **E** Base of Voltzberg, collecting event SR16-0316-01A, habitat of *T. sipaliwini* **F** Kasikasima, collecting event SR16-0324-01C, habitat of *T. sipaliwini*, *T. kasikasima*, *T. striatus*, and *T. emarginatus* sp. n. Red arrows point to example microhabitats where specimens were collected

The most frequently encountered species of the genus thus far in the eastern Guianas, *T. sipaliwini* has been found in a variety of seepage habitats, though most have been associated with more permanent flowing water (Fig. 16A–B, E–F). A few specimens have been taken along the margins of streams that were fed by or adjacent to rock seepages.

#### Tobochares striatus

Taxon classificationAnimaliaORDOFAMILIA

Short, 2013

Figs 2B , 5C–D , 8B , 9F , 10F , 11C , 12F , 14D , 15 , 16F

Tobochares striatus Short, 2013: 83.

##### Type material examined.

**Holotype** (male): “SURINAME: Sipaliwini District/ N2.24554°, W55.77000°, 800m/ Camp 2 Grensgebergte Rock/ leg. A. Short; rock seepages/ 12.iii.2012; SR12-0312-01A/ 2012 CI-RAP Survey” (NZCS). **Paratypes (11): SURINAME: Sipaliwini District**: Same data as type (3 exs., SEMC, one mounted on SEM stub); Camp 1, Upper Palumeu, 10.iii.2012, leg. A. Short, small forest pool by boulders, SR12-0310-02A (1 ex.; SEMC); Camp 4 (Kasikasima), 24.iii.2012, leg. A. Short, main seepage area, SR12-0324-01C (7 exs., SEMC, NZCS).

##### Additional material examined

**(1).** Camp 4 (Kasikasima), 24.iii.2012, leg. A. Short, main seepage area, SR12-0324-01C (1 ex., SEMC; DNA voucher SLE423).

##### Differential diagnosis.

See differential diagnosis for *T. kusad*.

##### Description.

***Size and form***: Body length 1.8–2.1 mm. Body elongate oval, moderately dorsoventrally compressed. ***Color and punctation*.** Dorsum of head very dark brown to black, anterolateral margins of clypeus with paler preocular patches (Fig. 5C–D); maxillary palps distinctly pale, with the distal half of palpomere 4 significantly darker. Pronotum brown to dark brown with the lateral margins appearing slightly paler (Fig. 8B); elytra brown to very dark brown, slightly paler at lateral margins and posteriorly (Fig. 2B). Meso- and metathoracic ventrites and abdominal ventrites very dark brown (nearly black), with prosternum slightly paler; epipleura, legs, labial palps, and antennae distinctly paler, with antennal club slightly darker than proximal antennal segments. Ground punctation on head, pronotum and elytra moderately fine. ***Head*.** Eyes measuring ~100µm anteroposteriorly, continuous with outline of head, emarginate at lateral margin, narrowing to half to slightly greater than half the width (Fig. 5C–D). ***Thorax*.** Elytra with ten rows of serial punctures which are depressed into grooves running the full length of the elytra (Fig. 11C). Metafemora mostly glabrous on ventral face, with narrow band of pubescence along proximal third of anterior margin (Fig. 12F). Elevation of mesoventrite forming a low transverse carina with an acute “tooth,” elevated to the same plane as the ventral surface of the mesocoxae (Fig. 9F). Metaventrite with distinct median ovoid glabrous area that is more than half of the total metaventrite length, and about half as wide as it is long (Fig. 10F). ***Abdomen*.** Abdominal ventrites uniformly and very densely pubescent, with small spicules interspersed amongst the setae (e.g. Fig. 13A). Aedeagus (Fig. 14D) with parameres slightly wider than median lobe; weakly parallel sided in basal third, then bulging slightly in middle third before tapering in apical third; apex of parameres very slightly expanded and bluntly rounded. Median lobe gradually tapering to a bluntly rounded apex, which slightly extends beyond the apex of the parameres; gonopore situated distinctly below the apex of the median lobe.

##### Distribution.

Known only from two localities in south-central Suriname (Fig. 15).

##### Biology.

Short (2013) noted that “most specimens were collected on a flowing seepage on granite (Fig. 16F). A single specimen was collected in a small forest pool near Camp 1 on the upper Palameu River, although this pool was situated directly beneath a group of large granite boulders.

#### Tobochares sulcatus

Taxon classificationAnimaliaORDOFAMILIA

Short & García, 2007

Figs 2A , 5E–F , 8C , 11F , 14B , 15 , 18A–B

Tobochares sulcatus Short & García, 2007: 4.

##### Material examined

**(100). VENEZUELA: Amazonas**: Tobogan de la Selva, 14.i.2009, leg. Short, García, Miller & Joly, wet rock covered with detritus, VZ09-0114-01F (50 exs., SEMC, MIZA, MALUZ; includes DNA vouchers SLE0035, SLE1027); same locality but 14.i.2009, leg. Short & Miller, partly shaded wet rock with algae, VZ09-0114-01G (2 exs., SEMC); same locality but 5.i.2006, leg. Short, wet rock with leaves, AS-06-011e (2 exs., SEMC includes DNA voucher SLE1037); same locality but 5.i.2006, leg. Short, pools in rock with sand, AS-06-011c (12 exs., SEMC); same locality but 16.xi.1987, leg. Spangler & Faitoute, Colln. #7 (1 ex., USNM); same locality but 26.i.1989, leg. Spangler, Faitoute, & Barr, seep at upper shelter (21, USNM); ca. 15 km S. Puerto Ayacucho, 13.ix.2007, leg. Short, AS-07-009a (3 exs., SEMC); nr. Iboruwa, “Tobogancito”, 13.i.2009, leg. Short et al., VZ09-0113-02X (1 ex., SEMC); nr. Hotel Nacamtur, 14.ix.2007, leg. Short, isolated rock seep with algae, AS-07-013x (1 ex., SEMC). **Bolivar**: Los Pijiguaos, seeps and stream on outcrop at night, 9.vii.2010, leg. Short et al., VZ10-0709-03A (2 exs., SEMC; includes one specimen on SEM stub and DNA voucher SLE1035); ca. 15 km N. Los Pijiguaos, 17.ix.2007, leg. Short & García, outcrop seepage, AS-07-016 (4 exs., SEMC); ca. 25 km E El Burro, 13.i.2009, leg. Short et al., rocky morichal, VZ09-0113-01X (1 ex., SEMC).

##### Differential diagnosis.

The dark brown coloration and deep elytral grooves, which are impressed along their entire length (Fig. 2A), separate this species from most other *Tobochares*, including *T. canaliculatus*, which also has deep grooves but is much paler and has a differently shaped aedeagus. Other congeners with impressed elytral grooves either have them only impressed on the posterior half of the elytra (e.g. *T. kasikasima*) or have very large serial punctures in those grooves (e.g. *T. striatus*, *T. kusad*), while the serial punctures themselves are minute to almost appearing absent in *T. sulcatus* (Fig. 11F).

##### Description.

***Size and form***: Body length 1.8–2.2 mm. Body elongate oval, moderately dorsoventrally compressed. ***Color and punctation*.** Dorsum of head very dark brown to black, anterolateral margins of clypeus with paler preocular patches (Fig. 5E–F); maxillary palps distinctly pale, with the apex of palpomere 4 darker (Fig. 8C). Pronotum brown to very dark brown with the lateral margins appearing slightly paler; elytra brown to very dark brown, slightly paler at lateral margins and posteriorly. Meso- and metathoracic ventrites and abdominal ventrites very dark brown (nearly black), with prosternum slightly paler; epipleura, legs, labial palps, and antennae distinctly paler, with antennal club slightly darker than proximal antennal segments. Ground punctation on head, pronotum and elytra moderately fine. ***Head*.** Eyes measuring ~100µm anteroposteriorly, continuous with outline of head, emarginate at lateral margin, narrowing to half to slightly more than half the width (Fig. 5E–F). ***Thorax*.** Elytra with ten rows of serial punctures which are depressed into deep, smooth grooves running the full length of the elytra. Metafemora mostly glabrous on ventral face, with narrow band of pubescence along proximal third of anterior margin. Elevation of mesoventrite forming a low transverse carina, not quite elevated to the same plane as the ventral surface of the mesocoxae. Metaventrite with distinct median ovoid glabrous area that is more than half of the total metaventrite length, and about half as wide as it is long. ***Abdomen*.** Abdominal ventrites uniformly and very densely pubescent, with small spicules interspersed amongst the setae (e.g. Fig. 13A). Aedeagus (Fig. 14B) with parameres about as wide as median lobe basally, parallel sided in basal half, then strongly narrowing in apical third; apex of parameres then broadly expanded and bluntly rounded. Median lobe gradually tapering to a bluntly rounded apex, which slightly extends beyond the apex of the parameres; gonopore situated distinctly below the apex of the median lobe.

##### Distribution.

Known from a series of localities along the northwestern edge of the Guiana Shield in Venezuela (Fig. 15), though the vast majority of material has been collected at Tobogan de la Selva.

##### Biology.

*Tobochares sulcatus* occurs on wet rocks with fallen leaves and other detritus along stream corridors (Fig. 18A–B). A handful of specimens have been collected from more isolated seepages, but these seem incidental compared to the long series—some in the hundreds—that have been found at the type locality Tobogan de la Selva. See Short and García (2007) for additional habitat details and images.

#### Tobochares

Taxon classificationAnimaliaORDOFAMILIA

sp. A

##### Material examined

**(1). VENEZUELA: Amazonas**: Tobogan de la Selva, leg. M. Balke (1 female, SEMC; DNA voucher SLE526).

##### Differential diagnosis.

This species is morphologically very similar to *T. pallidus*, and shares most diagnostic features of that species (in particular its very pale coloration). This species can be separated from *T. pallidus* by the faint but distinctly impressed striae in the posterior quarter of the elytra, and the slightly less emarginated eyes.

##### Remarks.

This species is only known from a single specimen, which we also extracted for DNA. Molecular data also supports this taxon as sister to *T. pallidus*, to which it is also similar morphologically. We refrain from describing the species until additional specimens, including ideally a male, can be found.

**Figure 17.**
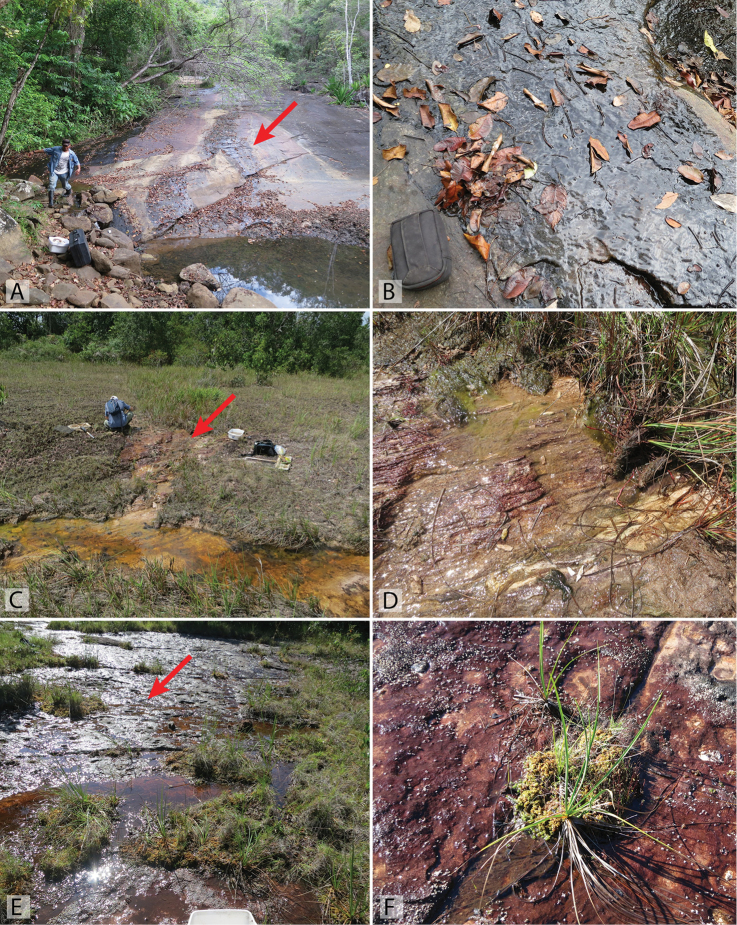
Habitat of *Tobochares* spp. in Guyana and Suriname. **A–B** Guyana, Kusad Mt., collecting event GY13-1027-03B, type locality for *T. kusad* sp. n. **C–D** Suriname, side of Kappel Airstrip, collecting event SR13-0824-02B, habitat of *T. kasikasima* **E–F** Suriname, summit of Tafelberg, Caiman Creek seepage, collecting event SR13-0819-01A, habitat of *T. kasikasima*. Red arrows point to example microhabitats where specimens were collected.

**Figure 18.**
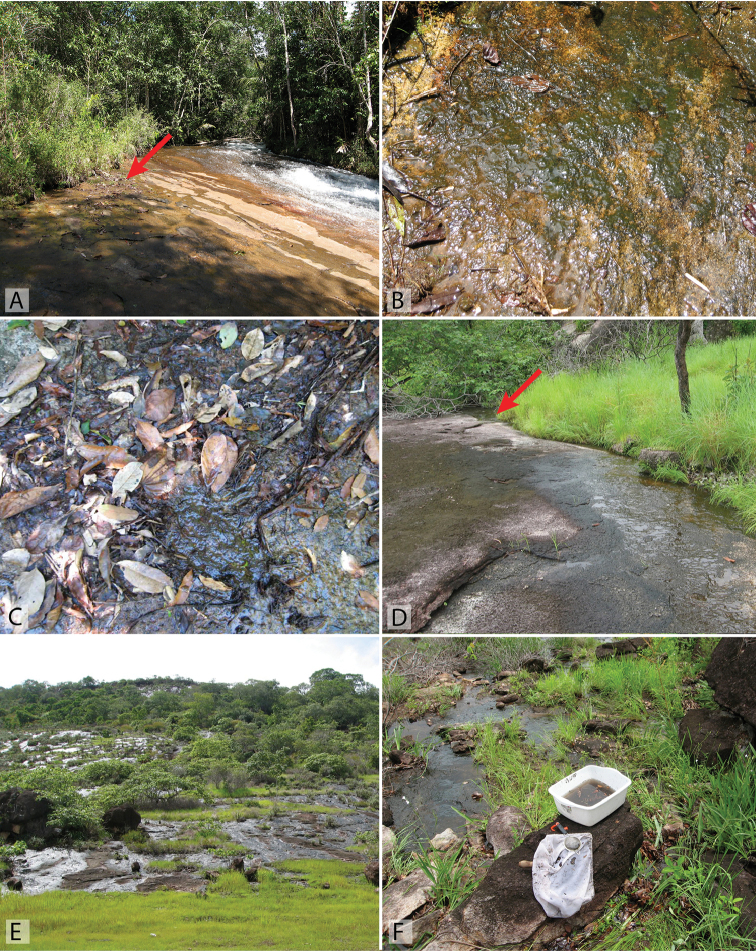
Habitat of *Tobochares* spp. in Venezuela. **A–B** Tobogan de la Selva, upstream, collecting event AS-08-080b, type locality for *T. canaliculatus* sp. n., *T. sulcatus*, *T. canthus* sp. n., and *T. pallidus* sp. n. **C** Tobogan de la Selva, collecting event VZ09-0114-01D **D** Outcrop near Pijiguaos, collecting event VZ10-0708-01B, habitat of *T. pallidus* sp. n. **E–F** Rock outcrop near Pijiguaos, collecting event VZ10-0709-01B, habitat of *T. pallidus* sp. n. Red arrows point to example microhabitats where specimens were collected.

### Key to the species of *Tobochares* Short & García

**Table:** 

1	Elytra with impressed grooves along their entire length (e.g. Figs 11C, E, F)	**2**
–	Elytra with impressed grooves in posterior half or less, or with grooves absent (e.g. Fig. 11A, B, D)	**5**
2	Apical maxillary palpomere uniformly pale (Fig. 8D). Pronotum and elytra light brown to brown, head brown, clypeus with large, distinctly pale preocular patches (Fig. 4A) (Venezuela)	***canaliculatus* sp. n.**
–	Apical maxillary palpomere darkened at least at apex, and sometimes on distal half or more (Fig. 8A–C). Pronotum and elytra brown to dark brown, head dark brown to black, clypeus with small, pale preocular patches	**3**
3	Punctures within elytral grooves small, grooves appearing fairly smooth (Fig. 11F). Elevation of mesoventrite forming transverse carina without tooth, not elevated to same plane as the ventral surface of the mesocoxae (Venezuela)	.***sulcatus* Short & García**
–	Punctures within elytral grooves strongly impressed and distinct (Fig. 11C). Elevation of mesoventrite forming transverse carina with tooth, elevated to same plane as the ventral surface of the mesocoxae	**4**
4	Apical maxillary palpomere with apex ranging from slightly to distinctly darkened (Fig. 8A). Eyes emarginate at lateral margin, narrowing to roughly two thirds the width (Fig. 5A–B) (Guyana)	***kusad* sp. n.**
–	Apical maxillary palpomere darkened in at least distal half (Fig. 8B). Eyes emarginate at lateral margin, narrowing to slightly greater than half the width (Fig. 5C–D) (Suriname)	***striatus* Short**
5	Elytra with grooves on posterior third or posterior half, grooves most prominent near elytral suture (e.g. Fig. 11A, B). Dorsum of head very dark brown to black, clypeus with faintly pale preocular patches (Fig. 4C, E). Elevation of mesoventrite forming trasverse carina with tooth, elevated to same plane as the ventral surface of the mesocoxae (Fig. 9D–E)	**6**
–	Elytra without grooves or with weak grooves on posterior quarter (e.g. Fig. 11D). Dorsum of head uniformly pale, or brown with distinctly pale preocular patches on clypeus (e.g. Fig. 6A, C, E). Elevation of mesoventrite forming low transverse carina without tooth, not elevated to same plane as the ventral surface of the mesocoxae (Fig. 9A, C)	**7**
6	Elytra with grooves on posterior half (Fig. 11A). Apical maxillary palpomere uniformly pale (Fig. 7A) (Guyana, Suriname)	***sipaliwini* Short & Kadosoe**
–	Elytra with grooves on posterior third (Fig. 11B). Apical maxillary palpomere with apex darkened (Fig. 7B) (Suriname)	***kasikasima* Short**
7	Dorsum of head, pronotum, and elytra uniformly pale (Fig. 3B); clypeus without pale preocular patches. Eyes emarginate at lateral margin, narrowing to about half of the width or slightly less (Fig. 6A–B) (Venezuela)	**8**
–	Dorsum of head, pronotum, and elytra brown to dark brown (e.g. Fig. 2C); clypeus with pale preocular patches. Eyes emarginate at lateral margin, narrowing to about a quarter of the width (Fig. 4C–F)	**9**
8	Elytra without grooves (Fig. 11D). Eyes emarginate at lateral margin, narrowing to about half of the width (Fig. 6A–B)	***pallidus* sp. n.**
–	Elytra with weak grooves on posterior quarter. Eyes emarginate at lateral margin, narrowing to slightly less than half of the width	**sp. A**
9	Aedeagus (Fig. 14H) with outer margin of parameres straight. Dorsal coloration light brown (Venezuela)	***canthus* sp. n.**
–	Aedeagus (Fig. 14I) with outer margin of parameres convex, tapering in anterior third. Dorsal coloration medium to dark brown (Suriname)	***emarginatus* sp. n.**

## Discussion

The water beetle seepage fauna of the Guiana Shield was essentially completely undescribed little more than 15 years ago. Since that time, numerous new lineages have been discovered in a variety of families, including Dytiscidae (e.g. *Fontidessus*), Noteridae (*Tonerus* Miller, 2009), and Hydroscaphidae (*Confossa* Short et al. 2015). In terms of Hydrophilidae, next to *Oocyclus*, species of *Tobochares* appear to be the dominant taxa in this habitat across the region.

Most sampled localities had only a single, or occasionally two species of *Tobochares* co-occurring in the same habitat. However, two particular sites had exceptional diversity with five and four species respectively: Tobogan de la Selva in Venezuela, and Mt. Kasikasima in Suriname. As there was no species composition overlap between them, a remarkable nine of the ten species covered in this revision could be collected by visiting just these two places.

Given how relatively little of the Guiana Shield has been surveyed, especially for hygropetric taxa, it is almost a certainty many more *Tobochares* species are left to be described.

## Supplementary Material

XML Treatment for Tobochares

XML Treatment for Tobochares canaliculatus

XML Treatment for Tobochares canthus

XML Treatment for Tobochares emarginatus

XML Treatment for Tobochares kasikasima

XML Treatment for Tobochares kusad

XML Treatment for Tobochares pallidus

XML Treatment for Tobochares sipaliwini

XML Treatment for Tobochares striatus

XML Treatment for Tobochares sulcatus

XML Treatment for Tobochares
